# Supplementary material for: Optimal Census by Quorum Sensing
Source: PLoS Comput Biol. 2015 May 12;11(5):e1004238. doi: 10.1371/journal.pcbi.1004238 (PMC4428632; doi:10.1371/journal.pcbi.1004238)
Supplement: S1 Text — The text contains detailed accounts of the model for quorum sensing, the formulation as a Gaussian channel, and the optimization of the quorum-sensing response. (PDF) [file pcbi.1004238.s001.pdf]

S1 Text

Thibaud Taillefumier[1] and Ned S. Wingreen[1,2]

[1] *Lewis-Sigler Institute for Integrative Genomics, Princeton University, Princeton, NJ 08544, USA*
[2] *Department of Molecular Biology, Princeton University, Princeton, NJ 08544, USA*

March 27, 2015

Contents

1 Stochastic model for quorum sensing **5**
- 1.1 Growth model 5
- 1.2 Internal dynamics of monitor protein abundance 7
- 1.3 External dynamics of autoinducer concentration 8

2 Quorum-sensing information channel **9**
- 2.1 Mean-field regime 10
- 2.2 Monitor output noise and Fano factor 12
- 2.3 Approximation for the mutual information 14

3 Optimal quorum sensing **16**
- 3.1 Solution to the Euler-Lagrange equations 16
- 3.2 Optimal quorum-sensing response 20
- 3.3 Optimal capacity of quorum sensing 28

Notation

$\boldsymbol{V}$: volume of the colony.

$\boldsymbol{\Omega}$: volume of the biomass of the colony.

$\boldsymbol{l}$: typical length scale of the colony.

$\boldsymbol{N}$: number of bacteria in the colony.

$\boldsymbol{\rho}$: cell density within the colony ($\rho = N/V$).

$\boldsymbol{\tau_d(\rho)}$: ρ-dependent bacterial division time.

$\boldsymbol{B_i}$: label of a bacterium in the colony.

$\boldsymbol{v_i}$: volume of bacterium B_i in the colony.

$\boldsymbol{\langle v \rangle}$: cellular volume averaged over the bacterial population.

$\boldsymbol{M_i}$: number of monitor proteins in bacterium B_i.

$\boldsymbol{r_p^{(i)}}$: rate of production of monitor proteins in bacterium B_i, in molecules per second.

$\boldsymbol{\tau_\delta}$: degradation time of the monitor proteins.

$\boldsymbol{\mu(\rho)}$: ρ-dependent metabolic coefficient modulating the production of proteins.

$\boldsymbol{\tau_m(\rho)}$: ρ-dependent lifetime of monitor proteins.

$\boldsymbol{m_i}$: concentration of monitor proteins in bacterium B_i.

$\boldsymbol{f_m(a, m)}$: output rate of monitor proteins, in molecules per second per cellular volume.

$\boldsymbol{f_{\mathrm{int}}}$: level of self-regulation of the monitor proteins.

$\boldsymbol{f_m^{(1)}(a)}$: base output rate of monitor proteins without internal feedback, in molecules per second per cellular volume.

$\boldsymbol{\sigma_m^{(i)}, \sigma_p^{(i)}}$: noise in monitor proteins due to production and degradation $\sigma_m^{(i)} = \sigma_p^{(i)}/v_i$.

$\boldsymbol{A}$: number of autoinducer molecules in the volume of the colony V.

$\boldsymbol{a}$: external concentration of autoinducer in the colony.

$\boldsymbol{r_a^{(i)}}$: rate of production of autoinducer molecules in bacterium B_i, in molecules per second.

$\boldsymbol{f_m(a,m)}$: output rate of monitor proteins, in molecules per second per cellular volume.

$\boldsymbol{D}$: diffusion coefficient of autoinducer molecules.

$\boldsymbol{\tau_a}$: time for autoinducers to escape from volume V by diffusion.

$\boldsymbol{f_{\text{ext}}(m)}$: output rate of autoinducer molecules, in molecules per second per cell.

$\boldsymbol{\sigma_a, \sigma_a^{(i)}}$: noise in monitor proteins due to production and escape.

$\boldsymbol{a_t}$: stochastic time course of the concentration of autoinducer.

$\boldsymbol{m_{i,t}}$: stochastic time course of the abundance of monitor proteins.

$\boldsymbol{a_t^{(\rho)}}$: stochastic time course of the concentration of autoinducer, at fixed cell density ρ.

$\boldsymbol{m_{i,t}^{(\rho)}}$: stochastic time course of the abundance of monitor proteins in bacterium B_i, at fixed cell density ρ.

$\boldsymbol{\overline{a}(\rho)}$: mean concentration of autoinducer at fixed cell density ρ.

$\boldsymbol{\overline{m}(\rho)}$: mean abundance of monitor proteins at fixed cell density ρ.

$\boldsymbol{\Sigma_m^2(\rho)}$: variance of the abundance of monitor proteins at fixed cell density ρ.

$\boldsymbol{F}$: Fano factor of monitor proteins, $F = \Sigma_m^2/\overline{m}$.

$\boldsymbol{D_{\text{TF}}, D_{\text{MP}}}$: diffusion coefficients of the transcription factors and the monitor proteins, respectively.

$\boldsymbol{l_{\text{TF}}, l_{\text{MP}}}$: typical linear sizes of the DNA targets of the transcription factors and the monitor proteins, respectively.

$\boldsymbol{\sigma_{\text{TF}}^2, \sigma_{\text{MP}}^2}$: spectral densities of the fluctuations in transcription factors and monitor proteins, respectively.

$\boldsymbol{\sigma_{\text{in}}}$: spectral density of the input noise in the production of monitor proteins.

$\boldsymbol{\sigma_{\text{out}}}$: spectral density of the output noise in the production of monitor proteins.

$\boldsymbol{b}$: burst size of monitor proteins.

$\boldsymbol{f_m^\infty}$: production rate of monitor proteins at autoinducer saturation.

$\boldsymbol{m_\infty}$: concentration of monitor proteins at saturation in the sRNA model of regulation, i.e. $m_\infty = \max\left(m_+, m_+/f_{\text{int},+}\right)$.

$\boldsymbol{(\rho_-, \rho_+)}$: range of cell densities over which quorum sensing is active.

$(\boldsymbol{a_-}, \boldsymbol{a_+})$: range of autoinducer concentrations.

$(\boldsymbol{m_-}, \boldsymbol{m_+})$: range of monitor protein concentrations over which quorum sensing is active.

$\boldsymbol{p(\rho)}$: distribution of cell densities.

$\boldsymbol{q(a)}$: distribution of autoinducer concentrations.

$\boldsymbol{q(m)}$: distribution of number of monitor proteins.

$\boldsymbol{I_{\rho,m}}$: mutual information between cell density and monitor proteins.

$\boldsymbol{\tilde{I}_{\rho,m}}$: small-noise expression for the mutual information between cell density and monitor proteins.

$\boldsymbol{\tilde{I}^{\star}_{\rho,m}}$: semi-numerical optimal small-noise mutual information between cell density and monitor proteins.

$\boldsymbol{I^{\star}_{\rho,m}}$: mutual information between cell density and monitor proteins for the optimal feedbacks.

$\boldsymbol{\tilde{C}_{\rho,m}}$: small-noise expression of the capacity of the quorum-sensing channel.

$\boldsymbol{\tilde{C}^{\star}_{\rho,m}}$: optimal small-noise capacity of the quorum-sensing channel.

$\boldsymbol{\tilde{C}_{a,m}}$: small-noise expression for the capacity of the detection channel.

$\boldsymbol{\tilde{C}^{\star}_{a,m}}$: optimal small-noise capacity of the detection channel.

In this supplement, we study theoretically the information about cell density available to a bacterium via quorum sensing. To do so, we adopt a slightly more general framework than in the main paper. In Section 1, we outline a model for the quorum-sensing response with feedbacks in a bacterial colony growing according to any specified time course. In Section 2, adopting biologically relevant approximations, we characterize the information channel associated with our quorum-sensing model. In Section 3, we optimize the information transfer of this information channel by varying the functions corresponding to the quorum-sensing feedbacks.

1 Stochastic model for quorum sensing

In this section, starting from simple biological considerations, we establish a dynamical model for the quorum-sensing response in a bacterial colony. In section 1.1, we adopt a simple model for bacterial growth. In section 1.2, we model the evolution of the abundance of a monitor protein (MP), which represents intracellularly the external concentration of autoinducer molecules (AIs). In section 1.3, we model the evolution of the external AI concentration during to the growth of a bacterial colony.

1.1 Growth model

Suppose that a bacterium B_0 founds a colony at time $t_0 = 0$. To model clonal growth in a volume V of typical length scale $l = 100\mu$m, we describe the stochastic occurrence of cell divisions as a birth process via the Markovian kinetic scheme:

$$B \xrightarrow{\tau_d} B + B\,. \tag{1}$$

For a rate of division $1/\tau_d$, such a scheme indicates that after division, each daughter cell divides independently after a random time distributed around the mean cell division period $T_d = \tau_d \ln 2$. Let us consider the successive times $t_1 < t_2 < \ldots$ of the division events occurring during clonal growth. We can then index cells according to their time of birth: at each time t_j, the dividing mother cell B_i, $i < j$, with cellular volume $v_i(t_j^-)$, splits into two daughter cells B_i and B_j, with respective cellular volumes $v_i(t_j^+)$ and $v_j(t_j^+)$:

$$B_i \xrightarrow{\tau_d} B_j + B_i\,, \quad \text{and} \quad v_i(t_j^-) = v_j(t_j^+) + v_i(t_j^+)\,. \tag{2}$$

At division time t_j, the number of monitor proteins (MPs) $M_{i,t}$ of the mother cells is allocated between the daughter cells:

$$M_{i,t_j^-} = M_{i,t_j^+} + M_{j,t_j^+}\,. \tag{3}$$

If the MPs are abundant in a bacterium, we can neglect the partition noise and we can assume that the number of proteins inherited from a mother cell is directly proportional

to its cell volume (if necessary partition noise can be included). As a result, the MP abundance, defined as the intracellular MP concentration $m_{i,t} = M_{i,t}/v_{i,t}$, is conserved at cell division:

$$m_{i,t_j^-} = \frac{M_{i,t_j^-}}{v_{i,t_j^-}} = \frac{M_{i,t_j^+} + M_{j,t_j^+}}{v_i(t_j^+) + v_j(t_j^+)} = \frac{M_{i,t_j^+}}{v_i(t_j^+)} = \frac{M_{j,t_j^+}}{v_j(t_j^+)} = m_{i,t_j^+} = m_{j,t_j^+} \,. \tag{4}$$

Moreover, between divisions, we can assume the cellular growth matches the rate of division so that the cellular volume of a cell B_i obeys for $t \geq t_i$

$$\frac{dv_i}{dt} = \frac{v_i}{\tau_d} \,, \tag{5}$$

with occasional discontinuities at division times. By conservation of cellular volumes, the overall biological volume $\Omega(t) = \sum_i v_i(t)$ is a continuous function satisfying the same first-order equation.

The above growth model can be generalized to the situation where the division time τ_d depends on the cell density $\rho = N/V$, where N is the number of cells in the volume V. For instance, over the growth period T, we consider the family of growth process parametrized by a growth exponent γ, which specifies the scaling of the rate of division with respect to the cell density:

$$\tau_d^{(\gamma)}(\rho) = \begin{cases} \dfrac{T}{\ln\left(\rho_+/\rho_-\right)} & \text{if} \quad \gamma = 0 \\[2ex] \dfrac{T\gamma\rho^{(\gamma)}}{\rho_+^{(\gamma)} - \rho_-^{(\gamma)}} & \text{if} \quad \gamma \neq 0 \end{cases} \tag{6}$$

over the cell density range (ρ_-, ρ_+). For each growth exponent γ, the biological volume follows a simple time course from $\Omega_- = \rho_- \langle v \rangle V$ to $\Omega_+ = \rho_+ \langle v \rangle V$

$$\Omega(t) = \begin{cases} \Omega_- \left(\dfrac{\Omega_+}{\Omega_-}\right)^{t/T} & \text{if} \quad \gamma = 0 \\[2ex] \Omega_- \left(1 + \left(\left(\dfrac{\Omega_+}{\Omega_-}\right)^{(\gamma)} - 1\right)\dfrac{t}{T}\right)^{1/\gamma} & \text{if} \quad \gamma \neq 0 \end{cases} \tag{7}$$

where $\langle v \rangle$ denotes the cellular volume averaged over the bacterial population. For $\gamma = 0$, the colony grows exponentially. For $\gamma \neq 0$, the population reaches high cell density either faster than exponentially when $\gamma > 0$, or slower than exponentially when $\gamma < 0$. Accordingly, the larger γ, the larger the fraction of time that bacteria spend at high cell density. We will only consider growth models that weakly deviate from exponential growth, i.e. $\gamma \approx 0$.

1.2 Internal dynamics of monitor protein abundance

Assuming large MP numbers, we adopt a continuous description for the MP molecular count $M_{i,t}$ and the MP abundance $m_{i,t}$ defined as the intracellular MP concentration. Specifically, we model the stochastic evolution of the MP abundance $M_{i,t}$ in the cell B_i via the Langevin equation

$$dM_{i,t} = \left(-\frac{M_{i,t}}{\tau_\delta} + r_p^{(i)}\right) dt + \sqrt{2}\sigma_p^{(i)}\, dW_t^{(i)}\,, \quad t > t_i\,, \tag{8}$$

where p/τ_δ is the rate of MP degradation, $r_p^{(i)}$ is the rate of MP expression, and where $dW^{(i)}$ is Gaussian white noise with coefficient $\sigma_p^{(i)}$. Both MP degradation rate and MP expression rate vary discontinuously at cell division. To simply account for these discontinuities of the MP production rate, we choose $r_p^{(i)}$ proportional to the cellular volume $v_i(t)$, adopting the form

$$r_p^{(i)} = v_i(t)\mu(\rho_t)f_m(a_t, m_{i,t}) \tag{9}$$

where f_m is the MP output rate per cellular volume and $\mu(\rho)$ is a metabolic coefficient. We assume that the metabolic coefficient scales with the growth rate as $\mu(\rho) = \tau_0/\tau_d(\rho)$, where τ_0 is the reference division time for exponential growth. The MP output rate f_m depends on the external AI concentration a_t and on the internal abundance of MPs $m_{i,t}$. The dependence on a_t models the regulation of MP expression by AI detection, while the dependence on $m_{i,t}$ models the self-regulation of MP expression by a memoryless internal feedback. Such a feedback can be established, e.g., via the fast binding of long-lived MPs to regulatory DNA regions that either promote or inhibit MP expression. Moreover, we assume that the MP output rate f_m is the product of a bare output rate function $f_m^{(1)}$ and the magnitude of self-regulation f_{int}

$$f_m(\rho, a, m) = f_m^{(1)}(a) f_{\text{int}}\,. \tag{10}$$

The dimensionless function f_{int} describes how self-regulation depends on the MP abundance m, e.g., via the occupancy of the regulatory DNA regions. The absence of self-regulation corresponds to $f_{\text{int}} = 1$, whereas $f_{\text{int}} < 1$ $(g > 1)$ indicates self-repression (self-induction). For simplicity, we also assume that the bare output rate function $f_m^{(1)}$ is a strictly increasing function of the AI concentration, which means that, in the absence of self-regulation ($f_{\text{int}} = 1$), the detection of AI molecules upregulates MP expression.

We can now specify the dynamics of monitor abundance $m_t = M_t/v(t)$. Using the dynamics of cellular growth (5), we deduce that

$$d\left(\frac{M_{i,t}}{v_i(t)}\right) = \frac{dM_{i,t}}{v_i(t)} - \frac{M_{i,t}}{v_i(t)}\frac{dt}{\tau_d}\,. \tag{11}$$

Then, from Eqs (8), (9), and (10), the stochastic evolution of the MP abundance $m_{i,t}$, $t \geq t_i$, obeys the Langevin equation

$$dm_{i,t} = \left(-\frac{m_{i,t}}{\tau_m(\rho_t)} + \mu(\rho_t)\, f_m(a_t, m_{i,t}) \right) dt + \sqrt{2}\sigma_m^{(i)}\, dW_t^{(i)} \tag{12}$$

where $1/\tau_m = 1/\tau_\delta + 1/\tau_d$ is the MP lifetime that may depend on ρ through τ_d. Without loss of generality, we only consider Eq. (12) for $\mu = 1$ since, for the specific models of noise later considered, all the results for $\mu \neq 1$ follow from the rescaling $\tau_m \to \mu\tau_m$. Moreover, as the MPs are generally very slowly degraded and the growth regime is roughly exponential, we assume that $\tau_m \approx \tau_d$. The stochasticity in MP abundance is modeled via independent Gaussian white noise $dW_t^{(i)}$ with noise coefficient $\sigma_m^{(i)} = \sigma_p^{(i)}/v_i(t)$ that may depend on the variables m_t and a_t through the MP degradation rate and the regulation of MP expression. In Section 2.2, we will justify the form of $\sigma_m^{(i)}$ as a simple function of the parameters and variables of the model.

1.3 External dynamics of autoinducer concentration

To model AI diffusion in the volume V, we adopt a continuous description for the number A_t of freely diffusing AI molecules. Denoting N_t the number of bacteria at time t, the stochastic evolution of A_t obeys the Langevin equation

$$dA_t = \left(-\frac{A_t}{\tau_a} + \sum_{i=0}^{N_t-1} r_a^{(i)} \right) dt + \sqrt{2 \sum_{i=0}^{N_t-1} \left(\sigma_a^{(i)}\right)^2}\, dW_t\,, \tag{13}$$

where $-A_t/\tau_a$ is the rate of AI loss by diffusion out of the volume and $r_a^{(i)}$ is the rate of AI production per cell. In the above equation, the stochasticity of the collective AI output is described by Gaussian white noise dW_t. The noise coefficient of dW_t follows from the scaling of AI-output noise for N_t independent cells

To account for the discontinuity in AI production at cell division, we assume that the AI output rate $r_a^{(i)}$ of bacterium B_i is proportional to the cell volume $v_i(t)$

$$r_a^{(i)} = \big(v_i(t)/\langle v \rangle\big)\, f_{\text{ext}}(m_{i,t})\,, \tag{14}$$

where $\langle v \rangle$ is the cellular volume averaged over the bacterial population. The function f_{ext} represents the AI output rate normalized by cell volume in molecules per cell per second, which depends on the internal MP abundance. The overall dependence of AI production on AI detection via the monitor abundance establishes an external feedback, which is a hallmark of quorum sensing.

For simplicity, we consider that the cell density of the colony ρ_t is well-approximated by its deterministic mean time course

$$\rho_t = \frac{N_t}{V} = \frac{\Omega(t)}{\langle v \rangle V}\,, \tag{15}$$

where the angular brackets denotes an average over the bacterial population. Such a simplification is valid for large populations of bacteria, e.g. $N > 100$. Moreover, averaging the changes in cellular volume over the cell cycle suggests a similar approximation for the overall AI production rate:

$$\sum_{i=0}^{N_t-1} r_a^{(i)} = \frac{1}{\langle v \rangle} \sum_{i=0}^{N_t-1} v_i(t) f_{\mathrm{ext}}(m_{i,t}) \approx N_t \Big\langle f_{\mathrm{ext}}(m_{i,t}) \Big\rangle_i . \tag{16}$$

Then, using Eq. (13) together with Eqs. (15) and (16), we can write the stochastic equation for the evolution of the external AI concentration a_t:

$$da_t = \left(-\frac{a_t}{\tau_a} + \rho_t \left\langle f_{\mathrm{ext}}(m_{i,t}) \right\rangle_i \right) dt + \sqrt{\frac{2\rho_t}{V}}\, \sigma_a \, dW_t \,, \quad \sigma_a^2 = \left\langle \left(\sigma_a^{(i)} \right)^2 \right\rangle_i , \tag{17}$$

where the cell density ρ_t is considered as an external deterministic driver.

In Eq. (17), the noise coefficient σ_a is intentionally left unspecified because the fluctuations in AI concentration play little role in quorum sensing. Indeed, for typical AI diffusion coefficients of order $D \approx 1000 \mu\mathrm{m}^2\mathrm{s}^{-1}$, AI molecules diffuse out of the volume V of typical length $l \approx 100\mu\mathrm{m}$ with timescale $\tau_a \approx l^2/D \approx 10\mathrm{s}$ [1]. Thus, there is a separation of timescales between the diffusion time τ_a and the lifetime τ_m of the MPs whose concentration tracks the changing cell density: $\tau_a \ll \tau_m \approx 30\mathrm{min}$. As a result, the AI concentration a_t can be considered as a fast variable which self-averages over the slow timescale of quorum sensing τ_m. Following [2], the adiabatic elimination of the fast variable prescribes the dynamics of AI concentration via simply balancing the rates of AI loss and production:

$$\frac{a_t}{\tau_a} = \rho_t \left\langle f_{\mathrm{ext}}(m_{i,t}) \right\rangle_i . \tag{18}$$

Eqs. (12) and (18) completely characterize the quorum-sensing response, i.e. the evolution of the external AI concentration and of the internal MP abundance, in response to bacterial growth.

2 Quorum-sensing information channel

In this section, we formulate the quorum-sensing system as a Gaussian information channel and quantify the information about cell density available to each bacterium. In section 2.1, we make a series of biologically motivated approximations to characterize quorum-sensing in the mean-field regime, for which we define the encoding scheme $p(\{m_i\}|\rho)$ of cell densities in terms of MP abundances. In section 2.2, we analyze the sources of noise in the quorum-sensing circuit to specify the encoding scheme $p\big(\{m_i\}|\rho\big)$ as a collection of Gaussian distributions. In section 2.3, we use the Gaussian approximation to express the mutual information (MI) between cell density and MP abundance in the small-noise approximation.

2.1 Mean-field regime

In order to assign an encoding scheme $p\big(\{m_i\}|\rho\big)$ to the quorum-sensing response, we consider the dynamical model defined by Eqs. (12) and (17) in the quasi-static and small noise approximation. In this approximation, the AI concentration a_t and the MP abundance $m_{i,t}$ evolve through a succession of Gaussian stationary states. These states are defined as the stationary measure of $\big(a_t^{(\rho)}, \{m_{i,t}^{(\rho)}\}\big)$, the linear-noise approximation of $\big(c, \{m_{i,t}\}\big)$ at fixed cell density ρ.

(i) The means $\big(\overline{a}(\rho), \overline{m}(\rho)\big)$ of the stationary process $\big(a_t^{(\rho)}, m^{(\rho)}\big)$ solve the self-consistent equations

$$\overline{a} = \tau_a \rho\, f_{\mathrm{ext}}(\overline{m}) \quad \text{and} \quad \overline{m} = \tau_m(\rho)\, f_m^{(1)}(\overline{a}) f_{\mathrm{int}}(\overline{m})\,. \tag{19}$$

At fixed ρ, the existence and uniqueness of solutions to (19) depend on the specifics of the functions τ_m, $f_m^{(1)}$, f_{ext}, and f_{int}. Here, we consider τ_m, $f_m^{(1)}$, f_{ext}, and f_{int} such that there exists a unique, strictly increasing cell density-MP abundance mapping $\overline{m}(\rho)$ that solves the self-consistent relation (19). This requirement discards quorum-sensing models where different cell densities are represented by the same internal MP abundance, which would prevent the faithful tracking of cell density.

(ii) At fixed ρ, i.e. at fixed cell count N, substituting a_t from Eq.(18) for in Eq. (12) reveals that we only have to consider the Gaussian dynamics of the N-dimensional vector $\big\{m_{i,t}^{(\rho)}\big\}$. The fluctuations $\delta m_{i,t}^{(\rho)} = m_{i,t}^{(\rho)} - \overline{m}(\rho)$ follow a linear stochastic equation of the form

$$d\left(\delta m_{i,t}^{(\rho)}\right) = -\sum_{j=0}^{n-1} \lambda_{i,j} \delta m_{i,t}^{(\rho)}\, dt + \sqrt{2}\sigma_m\, dW_t^{(i)}\,, \tag{20}$$

where σ_m is a shorthand notation for $\sigma_m^{(i)}$ evaluated at the operating point ρ, $\overline{a}(\rho)$, and $\overline{m}(\rho)$. The sensitivities $\lambda_{i,j}$ takes the value λ_1 on the diagonal and the value λ_2 off the diagonal, computed as:

$$\lambda_1 = \frac{1}{\tau_m} - f_m^{(1)}(\overline{a}) g'(\overline{m}) - \frac{\tau_a}{V} f'_{\mathrm{ext}}(\overline{m}) f_m^{(1)\prime}(\overline{a}) f_{\mathrm{int}}(\overline{m})\,, \tag{21}$$

$$\lambda_2 = -\frac{\tau_a}{V} f'_{\mathrm{ext}}(\overline{m}) f_m^{(1)\prime}(\overline{a}) f_{\mathrm{int}}(\overline{m})\,. \tag{22}$$

The stability of the mean value $\overline{m}(\rho)$ imposes the positivity of the eigenvalues of the sensitivity matrix, i.e. $\mu_1 = \lambda_1 - \lambda_2 > 0$ and $\mu_2 = \lambda_1 + (N-1)\lambda_2 > 0$. Under these constraints, we compute the stationary variance $\Sigma_m^2(\rho) = \big\langle \delta m_{i,t}^{(\rho)} \delta m_{i,t}^{(\rho)} \big\rangle$ and the stationary

covariance $C_m(\rho) = \langle \delta m_{i,t}^{(\rho)} \delta m_{j,t}^{(\rho)} \rangle$ as:

$$\Sigma_m^2(\rho) = \frac{\sigma_m^2}{\lambda_1 - \lambda_2}\left(1 - \frac{\lambda_2}{\lambda_1 + (N-1)\lambda_2}\right), \tag{23}$$

$$C_m(\rho) = -\frac{\sigma_m^2 \lambda_2}{(\lambda_1 - \lambda_2)\big(\lambda_1 + (N-1)\lambda_2\big)}. \tag{24}$$

We can verify that the variance $\Sigma_m^2(\rho)$ is necessarily positive. By contrast, for an increasing bare output rate $f_m^{(1)}$, the covariance $\Sigma_m^2(\rho)$ is positive (negative) for an increasing (decreasing) AI output rate f_{ext}.

We now make an important biologically motivated approximation. As quorum sensing takes place in large bacterial colonies, we consider that the external AI concentration, seen as a mean-field signal, averages out over the population of cells. In this approximation, bacteria sample independently the deterministic AI concentration $\overline{a}(\rho)$. Together with the quasi-stationary and small-noise approximation, this defines the mean-field regime of quorum sensing. For such a regime, the covariance between the MP abundance in different bacteria vanishes $C_m(\rho) \to 0$, and the variance $\Sigma_m^2(\rho)$ simplifies to:

$$\Sigma_m^2(\rho) \to \frac{\tau_m \sigma_m^2}{1 - \tau_m f_m^{(1)}(\overline{a}) g'(\overline{m})}. \tag{25}$$

Thus, the encoding scheme $p\big(\{m_i\}|\rho\big)$ of the information channel associated with our quorum-sensing model is specified via the independent Gaussian distributions $p(m_i|\rho) \sim \mathcal{N}\big(\overline{m}(\rho), \Sigma_m(\rho)\big)$. This encoding scheme depends on the AI output rate f_{ext} and the self-regulation function f_{int}, which respectively establish the external feedback and the internal feedback of the quorum-sensing circuit. Instead of manipulating directly these feedback functions, it is actually convenient to work with cell density-AI concentration mapping $\overline{a}(\rho)$ and the cell density-MP abundance $\overline{m}(\rho)$. This requires a slightly altered expression for $\Sigma_m^2(\rho)$. Differentiating the self-consistent relation (19) with respect to m, substituting for f_{int} in expression (25), and remembering that a and m are implicitly functions of ρ, leads to

$$\Sigma_m^2(\rho) = \frac{\tau_m \overline{m}'(\rho)\sigma_m^2}{f_{\text{int}}(\overline{m})\partial_\rho\left[\tau_m f^{(1)}(\overline{a})\right]}. \tag{26}$$

Thus, we have replaced the variance's dependence on the feedback functions f_{ext} and f_{int} by a dependence on the cell density-AI concentration mapping $\overline{a}(\rho)$ and the cell density-MP abundance mapping $\overline{m}(\rho)$. This change of variables is legitimate as there is a one-to-one correspondence between the functions (f_{ext}, g) and the mappings $(\overline{a}, \overline{m})$, through the self-consistent relations (19).

2.2 Monitor output noise and Fano factor

To further characterize the encoding scheme $p(m_i|\rho) \sim \mathcal{N}\big(\overline{m}(\rho), \Sigma_m(\rho)\big)$ of our quorum-sensing model, we need to determine the functional form of the MP noise function σ_m. This requires to specify the different sources of noise and to estimate their contributions to the stochasticity in MP abundance. Here, we show that such an analysis for biologically relevant quorum-sensing models yields noise functions σ_m that are simply expressed in terms of the well-studied Fano factor. Noise in gene expression comes in two forms, which we discuss in paragraph (i) and (ii):

(i) The input noise σ_{in} quantifies the variability in MP expression due to the propagation of the input's fluctuations through the gene expression machinery. For a self-regulating gene, we consider the concentration c_{TF} of an active transcription factor (TF) and the MP concentration m as inputs. The randomness in the diffusive arrivals of TFs and MPs at their targets gives rise to fluctuations in the TF concentration and in the MP concentration. Such fluctuations can be modeled as the results of independent white noise contributions with respective spectral densities denoted as σ^2_{TF} and σ^2_{MP}. At fixed cell density ρ, both these spectral densities have been shown to be given accurately via simple dimensional analysis [3]:

$$\sigma^2_{\text{TF}} = \frac{\overline{a}_{\text{TF}}(\rho)}{D_{\text{TF}} l_{\text{TF}}} \quad \text{and} \quad \sigma^2_{\text{MP}} = \frac{\overline{a}_{\text{MP}}(\rho)}{D_{\text{MP}} l_{\text{MP}}}\,, \tag{27}$$

where $\overline{a}_{\text{TF}}$ is the mean concentration of active TF, D_{TF} and D_{MP} are diffusion coefficients, and l_{TF} and l_{MP} are linear sizes of target DNA sequences. The fluctuations in TF and MP due to diffusive arrivals propagate through the MP expression machinery, yielding in the small-noise approximation

$$\sigma^2_{\text{in}} = \big(\partial_{\overline{a}_{\text{TF}}} f_m\big)^2 \sigma^2_{\text{TF}} + \big(\partial_{\overline{m}} f_m\big)^2 \sigma^2_{\text{MP}} \tag{28}$$

where $f_m = f_m^{(1)} g$ is the MP output rate per cellular volume. We consider that, in the absence of feedback, the bare output rate is proportional to the mean concentration of active TF: $f_m^{(1)} = k_{\text{TF}} \overline{a}_{\text{TF}}$. Then, using the self-consistent relation (19), the input noise satisfies

$$\sigma^2_{\text{in}} = \left(\frac{k_{\text{TF}} f_{\text{int}}(\overline{m})}{\tau_m D_{\text{TF}} l_{\text{TF}}} + \frac{\big(f_m^{(1)}(\overline{a}) g'(\overline{m})\big)^2}{D_{\text{MP}} l_{\text{MP}}} \right) \overline{m}\,. \tag{29}$$

(ii) The output noise σ_{out} quantifies the variability in MP expression due to the inherent stochasticity of molecular processes at fixed input. In our "TF regulation" model, AI molecules induce the production of MPs by allosterically regulating the TF, which only binds to its cognate DNA regulatory sequence when complexed with AI. In the more complex "sRNA regulation" model, the TF positively regulates a small regulatory RNA

(sRNA) that represses MP expression [4]. In the presence of AI, the formation of AI-TF complexes prevents the TF from binding to its cognate promoter DNA sequence, thus inhibiting sRNA expression and allowing MP expression. For strong sRNA-mRNA pairing in the expressed regime, the mean MP output rate can have the same form f_m in the TF regulation model and in the sRNA regulation model [5]. For clarity, we recall the sets of Langevin equations that model TF regulation and RNA regulation using the same notations as in [5], where s, m, and p denote the intracellular sRNA abundances , the mRNA abundance, and protein abundance, respectively:

$$\text{TF}: \quad \begin{aligned} \frac{dm}{dt} &= \alpha_m - \beta_m m + \xi_m\,, \\ \frac{dp}{dt} &= \gamma_m - \beta_p p + \xi_p\,, \end{aligned} \qquad \text{sRNA}: \quad \begin{aligned} \frac{ds}{dt} &= \alpha_s - \beta_s s - ksm + \xi_s + \xi_k\,, \\ \frac{dm}{dt} &= \alpha_m - \beta_m m - ksm + \xi_m + \xi_k\,, \\ \frac{dp}{dt} &= \gamma_m - \beta_p p + \xi_p\,, \end{aligned} \tag{30}$$

In the above equations, α_s and α_m are transcriptional rates, γ_m is the translational rate, β_s, β_m, β_p are degradation/dilution rates, and k is the kinetic constant between sRNA and mRNA. In particular, the burst size $b = \gamma_m/\beta_m$. The molecular Poisson noise associated with each reaction is modeled via independent white noise ξ_s, ξ_m, ξ_p, and ξ_k satisfying $\langle \xi_s^2 \rangle = \alpha_s + \beta_s \langle s \rangle$, $\langle \xi_m^2 \rangle = \alpha_m + \beta_m \langle m \rangle$, $\langle \xi_k^2 \rangle = k \langle s \rangle \langle m \rangle$, and $\langle \xi_p^2 \rangle = \gamma_m \langle m \rangle + \beta_p \langle p \rangle$. Reverting to our notations, Langevin analysis at steady state reveals that the output noise σ_{out} differs for the two model. Solving the Langevin equation for TF regulation yields the noise function

$$\sigma_{\text{out}}^2 = (1+b) f_m / \langle v \rangle\,, \tag{31}$$

where $\langle v \rangle$ is the cellular volume averaged over the bacterial population and where the burst size b is the average number of translated MPs per transcript [6]. By contrast, assuming that the sRNAs only weakly interact with any other targets, the output noise for the sRNA regulation is

$$\sigma_{\text{out}}^2 = \left(1 + b \frac{f_m}{f_m^\infty}\right) \frac{f_m}{\langle v \rangle}\,, \tag{32}$$

where f_m^∞ is the MP output rate at saturation, i.e. when the sRNA expression is silenced [7]. Thus, in the low expression regime ($f_m < f_m^\infty$), sRNA regulation reduces the MP mRNA lifetime, yielding an effective burst size $b f_m / f_m^\infty$ that is smaller than the burst size b for TF regulation. As a result of this smaller effective burst size, the stochasticity in MP expression is reduced.

We can now compare the respective contributions of the input noise σ_{in}^2 and the output noise σ_{out}^2 for biologically relevant values of the model's parameters. The typical diffusion coefficient for a protein is $D \approx 10 \mu\text{m}^2 s^{-1}$ and the typical size of a bacterial DNA regulatory

sequence is a few tens of nucleotides ($l \approx 66$nm for 20 nucleotides) [8, 9]. Measurements of the quorum-sensing response in *V. fisheri* and *V. harveyi* implies long-lived abundant MPs ($\tau_m \approx 30$min, $m_- \approx 100\mu\text{m}^{-3}$, $m_- \approx 600\mu\text{m}^{-3}$), a large total number of TFs (≈ 100 copies per bacterium), and a weak graded self-regulation ($f_{\text{int},-} \approx 2$, $f_{\text{int},+} \approx 1/2$) [10, 11]. Assuming all TFs are activated at high cell density ($a_{\text{TF}} \approx 100\mu\text{m}^{-3}$), we deduce from the above values that $k_{\text{TF}} \approx 1/150\text{s}^{-1}$ and $f'_{\text{int}} \approx 1/250\mu\text{m}^3$. In turn, this implies that, for a burst size $b = 20$, we have $\sigma_{\text{in}}^2/\sigma_{\text{out}}^2 \leq 0.02$ for the low expression regime of sRNA regulation and $\sigma_{\text{in}}^2/\sigma_{\text{out}}^2 \leq 0.002$ for the high expression regime of sRNA regulation and for the whole regime of TF regulation. Therefore, for biologically relevant parameters, the noise coefficient σ_m is well approximated by the output noise σ_{out}. Moreover, expressions (31) and (32) reveal that σ_m can be written as a function of the MP lifetime τ_m and the mean MP abundance $\overline{m}$. Indeed, in the quasi-static approximation, $f_m = \overline{m}/\tau_m$, which leads to

$$\text{TF} : \sigma_m^2 = \frac{(1+b)\overline{m}}{\tau_m \langle v \rangle} \qquad \text{sRNA} : \sigma_m^2 = \frac{(1 + b\,\overline{m}/m_\infty)\,\overline{m}}{\tau_m \langle v \rangle}, \tag{33}$$

where m_∞ is the MP abundance when the sRNA is not expressed. More generally, the above analysis suggests that one should characterize the noise coefficient σ_m via a function that simply relates the fluctuations[1] of MP expression $\big(\Sigma_m^{(1)}\big)^2$ to the mean MP abundance $\overline{m}$. Such a function is conveniently given by the Fano factor $F^{(1)}(\overline{m}) = \big(\Sigma_m^{(1)}\big)^2/\overline{m}$ of MP expression, which has been studied theoretically and experimentally for a number of biological systems. Notably, $F^{(1)} = 1$ for Poisson noise. Then, for a bacterial colony of density ρ, we can write the noise coefficient σ_m in its most general form as

$$\sigma_m = \sqrt{\frac{\overline{m}(\rho) F^{(1)}\big(\overline{m}(\rho)\big)}{\tau_m(\rho)}}, \tag{34}$$

where the Fano factor may now comprise other sources of noise, for instance fluctuations due to the environment or other intracellular factors.

2.3 Approximation for the mutual information

Given a functional form for the noise coefficient σ_m, the encoding scheme $p\big(\{m_i\}|\rho\big)$ fully characterizes the quorum-sensing information channel associated with our model. To quantify the information transferred by the information channel, we need an explicit formula for the MI between cell density ρ and MP abundance m. This requires us to specify the input distribution of cell densities, which is determined by bacterial growth. If we assume a growth function Ω varying from Ω_- to Ω_+ over a total time course T, we can infer the

[1] Here, $\big(\Sigma_m^{(1)}\big)^2$ is to be understood as the fluctuations of MP abundance at fixed AI concentration and without internal feedback.

corresponding distribution of cell densities $\rho = \Omega/(\langle v\rangle V)$ on (ρ_-, ρ_+) that would result from independent uniform sampling in time:

$$p(\rho) = \frac{\langle v\rangle V}{T\Omega'(t)} = \frac{\langle v\rangle V}{T\Omega'\big(\Omega^{-1}(\rho\langle v\rangle V)\big)}\,. \tag{35}$$

For the family of growth functions defined by Eq. (7), the input distribution of cell densities satisfies $p(\rho) \propto \rho^{\gamma-1}$ on (ρ_-, ρ_+).

Employing this model for the input distribution p, the MI between cell density and AI concentration can be written

$$I_{\rho,m} = H_m - \int_{\rho_-}^{\rho_+} H(m\,|\,\rho)p(\rho)\,d\rho\,, \tag{36}$$

where H_m is the entropy of the unconditioned MP abundance m and where $H(m\,|\,\rho)$ is the conditional entropy of m knowing that the population density is ρ [12]. Since the encoding scheme $p\big(\{m_i\}|\rho\big)$ is dictated by independent Gaussian distributions $\mathcal{N}\big(\overline{m}(\rho), \Sigma_m^2(\rho)\big)$, we have simply

$$H(m\,|\,\rho) = \frac{1}{2}\log_2(2\pi e\Sigma_m^2(\rho))\,, \tag{37}$$

while H_m is determined by the output distribution of MP abundance $q(m)$ as the convolution of $p(\rho)$ with the Gaussian distribution $\mathcal{N}\big(\overline{m}(\rho), \Sigma_m^2(\rho)\big)$.

In the small-noise approximation, the fluctuations of the conditional concentration $m^{(\rho)}$ are small with respect to its mean value $\Sigma_m(\rho) \ll \overline{m}(\rho)$ [13]. In this approximation, it is valid to approximate the output distribution $q(m)$ as the transform of the input distribution $p(\rho)$ through the deterministic mapping $\overline{m}(\rho)$. This yields a lower bound for the entropy

$$H_m = -\int_{m_-}^{m_+} q(m)\log_2\big(q(m)\big)\,dm \simeq -\int_{\rho_-}^{\rho_+} p(\rho)\log_2\left(\frac{p(\rho)}{\overline{m}'(\rho)}\right)d\rho\,,$$

which is also the leading term in a small-noise expansion. We find for the small-noise approximation of the MI

$$\begin{aligned}\tilde{I}_{\rho,m} &= -\int_{\rho_-}^{\rho_+} p(\rho)\log_2\left(\frac{p(\rho)}{\overline{m}'(\rho)}\right)d\rho - \frac{1}{2}\int_{\rho_-}^{\rho_+} p(\rho)\log_2\big(2\pi e\,\Sigma_m^2(\rho)\big)\,d\rho\,,\\ &= H_\rho + \int_{\rho_-}^{\rho_+} p(\rho)\log_2\left(\frac{1}{\sqrt{2\pi e}\,\delta_\rho}\right)d\rho\,,\end{aligned} \tag{38}$$

where

$$H_\rho = -\int_{\rho_-}^{\rho_+} d\rho\, p(\rho)\log_2 p(\rho)\,, \tag{39}$$

is the entropy of $p(\rho)$. The ratio $\delta_\rho = \Sigma_m(\rho)/\overline{m}'(\rho)$, referred to as the "resolution" of the quorum-sensing channel, quantifies the smallest difference in cell density that a bacterium can resolve by reading out its MP abundance. In order to express the MI $\tilde{I}_{\rho,m}$ as a functional of the cell density-AI concentration mapping $\overline{a}(\rho)$ and of the cell density-MP abundance mapping $\overline{m}(\rho)$, we use relation (26) to express the squared resolution of the quorum-sensing channel as

$$\delta_\rho^2 = \left(\frac{\Sigma_m(\rho)}{\overline{m}'(\rho)}\right)^2 = \left(\frac{\partial_\rho\big[\tau_m(\rho)\, f_m^{(1)}\big(\overline{a}(\rho)\big)\big]}{\tau_m(\rho)\, f_m^{(1)}\big(\overline{a}(\rho)\big)}\, \frac{\overline{m}'(\rho)}{F\big(\overline{m}(\rho)\big)}\right)^{-1} . \tag{40}$$

Thus, optimizing the small-noise MI $\tilde{I}_{\rho,m}$ over the mappings $\overline{a}(\rho)$ and $\overline{m}(\rho)$, i.e. over the feedback functions f_{ext} and f_{int}, appears as a problem in the calculus of variations with the action-like functional:

$$\begin{aligned} \tilde{I}_{\rho,m}[\overline{a},\overline{m}] &= H_\rho - \frac{1}{2}\log_2(2\pi e) \\ &+ \int_{\rho_-}^{\rho_+} \frac{p(\rho)}{2} \log_2\left(\frac{\partial_\rho\big[\tau_m(\rho)\, f_m^{(1)}\big(\overline{a}(\rho)\big)\big]}{\tau_m(\rho)\, f_m^{(1)}\big(\overline{a}(\rho)\big)}\, \frac{\overline{m}'(\rho)}{F\big(\overline{m}(\rho)\big)}\right) d\rho\,. \end{aligned} \tag{41}$$

3 Optimal quorum sensing

In this section, we specify the quorum-sensing circuit that optimizes the MI $\tilde{I}_{\rho,m}$ between the cell density ρ and the MP abundance m, by varying the internal and external feedbacks f_{ext} and f_{int}. In Section 3.1, we solve the Euler-Lagrange equations associated with optimizing $\tilde{I}_{\rho,m}$ as a problem in the calculus of variations. In Section 3.2, we specify the response of the optimal quorum-sensing system and we discuss the role of the feedbacks. In Section 3.3, we show that the optimal quorum-sensing system, as an information channel, operates at capacity.

3.1 Solution to the Euler-Lagrange equations

In the small-noise approximation, optimizing the MI $\tilde{I}_{\rho,m}$ of our quorum-sensing system has the form of a variational problem in classical mechanics. In particular, for a given sensory circuit specified by (f_m, σ_m), optimizing the MI $\tilde{I}_{\rho,m}$ with respect to $\overline{a}$ and $\overline{m}$ for a given input distribution p, corresponds to optimizing the action-like functional

$$\mathcal{A}[\overline{a},\overline{m}] = \int_{\rho_-}^{\rho_+} \Big(\mathcal{L}_a\big[\overline{a}'(\rho),\overline{a}(\rho),\rho\big] + \mathcal{L}_m\big[\overline{m}'(\rho),\overline{m}(\rho),\rho\big]\Big)\, d\rho\,. \tag{42}$$

where the Lagrangians $\mathcal{L}_a$ and $\mathcal{L}_m$ are respectively the $\overline{a}$-dependent and the $\overline{m}$-dependent parts of expression (41):

$$\mathcal{L}_a\big[\overline{a}',\overline{a},\rho\big] = p(\rho)\log_2\left(\frac{\partial_\rho\big[\tau_m(\rho)f_m^{(1)}(\overline{a})\big]}{\tau_m(\rho)f_m^{(1)}(\overline{a})}\right) \tag{43}$$

$$\mathcal{L}_m\big[\overline{m}',\overline{m},\rho\big] = p(\rho)\log_2\left(\frac{\overline{m}'}{F^{(1)}(\overline{m})}\right). \tag{44}$$

The sample space $\{\overline{a},\overline{m}\}$ over which we will optimize the action $\mathcal{A}$ consists of all the twice-differentiable functions with boundary conditions $\overline{a}(\rho_-)=a_-$, $\overline{a}(\rho_+)=a_+$, $\overline{m}(\rho_-)=m_-$, $\overline{m}(\rho_+)=m_+$ and for which the positive mappings $\tau_m(\rho)f^{(1)}[\overline{a}(\rho)]$ and $\overline{m}(\rho)$ are strictly increasing with respect to the cell density. In the context of the Lagrangian formalism, note that ρ plays the role of the time index, while $(\overline{a},\overline{m})$ is equivalent to a ρ-dependent path connecting (a_-,m_-) to (a_+,m_+).

The stationary path $(\overline{a}^\star,\overline{m}^\star)$ that locally extremizes the action $\mathcal{A}[\overline{a},\overline{m}]$ solves the Euler-Lagrange equations:

$$\frac{\partial\mathcal{L}_a}{\partial\overline{a}}-\frac{d}{d\rho}\left(\frac{\partial\mathcal{L}_a}{\partial\overline{a}'}\right)=0 \quad\text{and}\quad \frac{\partial\mathcal{L}_m}{\partial\overline{m}}-\frac{d}{d\rho}\left(\frac{\partial\mathcal{L}_m}{\partial\overline{m}'}\right)=0\,. \tag{45}$$

Both Euler-Lagrange Eqs. (45) admit simple analytical formulas for their stationary path $a^\star$ and $m^\star$. Indeed, these equations can be integrated with respect to ρ to yield the following first integrals of the problems

$$\frac{\partial_\rho\big[\tau_m f_m^{(1)}(\overline{a})\big]}{\tau_m f_m^{(1)}(\overline{a})}=Z_a\,p(\rho) \quad\text{and}\quad \frac{\overline{m}'(\rho)}{F\big(\overline{m}(\rho)\big)}=Z_m\,p(\rho)\,. \tag{46}$$

where Z_a are Z_m are constants. It is important to observe that, by positivity of the input distribution $p(\rho)$, the first integral relations (46) impose that the stationary mappings $\overline{m}^\star$ and $\tau_m(\rho)f_m^{(1)}[\overline{a}(\rho)]$ are strictly increasing with respect to the cell density ρ, and thus positive. In turn, we integrate these relations to obtain the equation satisfied by the stationary path $\big(a^\star(\rho),m^\star(\rho)\big)$ in terms of the cumulative input distribution $P(\rho)$

$$\ln\Big(\tau_m(\rho)f_m^{(1)}(\overline{a})f_{\text{int},-}/m_-\Big)=Z_aP(\rho) \quad\text{and}\quad Z(\overline{m})=Z_mP(\rho)\,, \tag{47}$$

where $P(\rho)=\int_{\rho_-}^{\rho_+}p(\rho)\,d\rho=t/T$ and where $Z(m)=\int_{m_-}^{m}dm/F^{(1)}(m)$. Finally, the normalization condition $P(\rho_+)-P(\rho_-)=1$ prescribes the constants Z_a and Z_m that appear in the the first integrals of the problem

$$Z_a=\ln\left(\frac{m_+f_{\text{int},-}}{m_-f_{\text{int},+}}\right) \quad\text{and}\quad Z_m=\int_{m_-}^{m_+}\frac{dm}{F^{(1)}(m)}\,. \tag{48}$$

From Eqs. (47) and (48), we can deduce a closed-form expression for $\overline{a}^\star$ and $\overline{m}^\star$, both being increasing functions of the cell density ρ. Eqs. (44) and (46) yield the stationary value of the MI $I_{\rho,m}[\overline{a}^\star, \overline{m}^\star] = \tilde{I}^\star_{\rho,m}$:

$$\tilde{I}^\star_{\rho,m} = \frac{1}{2}\log_2\left(\frac{Z_a Z_m}{2\pi e}\right) = \frac{1}{2}\log_2\left(\ln\left(\frac{m_+ f_{\text{int},-}}{m_- f_{\text{int},+}}\right)\int_{m_-}^{m_+} \frac{dm}{2\pi e\, F^{(1)}(m)}\right). \tag{49}$$

We still have to demonstrate that the stationary path is an optimal path, i.e. that $\tilde{I}^\star_{\rho,m}$ is the global maximum of the MI $\tilde{I}_{\rho,m}$ over the sample space $\{\overline{a}, \overline{m}\}$. To do so, we just need to recognize that from a mathematical standpoint, the change of variables

$$u(\rho) = \ln\left[\tau_m(\rho) f_m^{(1)}\big(\overline{a}(\rho)\big)\right] \quad \text{and} \quad v(\rho) = \int_{m_-}^{\overline{m}(\rho)} \frac{dm}{F^{(1)}(m)} \tag{50}$$

are legitimate one-to-one mappings that sends the sample space $\{\overline{a}, \overline{m}\}$ onto the space $\{u, v\}$ of twice-differentiable increasing functions $\{u, v\}$ such that $u(\rho_-) = v(\rho_-) = 0$ and such that $u(\rho_+) = Z_a$ and $v(\rho_+) = Z_m$. The transformed sample space $\{u, v\}$ is a convex set. Furthermore, implementing the change of variables (50) in Eqs. (43) and (44) yields a simple action-like functional of the form:

$$\mathcal{A}[u, v] = \int_{\rho_-}^{\rho^+} p(\rho)\big[\log_2 u'(\rho) + \log_2 v'(\rho)\big]\, d\rho\,. \tag{51}$$

The positivity of the distribution $p(\rho)$ and the concavity of the $\log_2$ function ensures that the action $\mathcal{A}[u, v]$ is concave in its arguments. By concavity of the action $\mathcal{A}\big[u, v\big]$ and convexity of the set $\{u, v\}$, the stationary path $(u^\star, v^\star)$, that is obtained from $(\overline{a}^\star, \overline{m}^\star)$ by the change of variables (50), is an optimal path of $\mathcal{A}$. This establishes that the path $(\overline{a}^\star, \overline{m}^\star)$ globally optimizes the MI $\tilde{I}_{\rho,m}$ over $\{\overline{a}, \overline{m}\}$, showing that $\tilde{I}^\star_{\rho,m}$ is the optimal information transfer of our quorum-sensing model in the small-noise approximation.

The optimization of the MI $\tilde{I}_{\rho,m}$ as a problem in the calculus of variations allows us to fully characterize the optimal quorum-sensing response in the small-noise approximation. Before discussing this optimal response for our two examples of MP regulation, we first give explicit formulas for various optimal quantities.

Optimal AI concentration and MP abundance mappings

Eqs. (47), together with the fact that $Z(m)$ and $f_m^{(1)}(a)$ are strictly increasing functions yields the optimal cell density-AI concentration mapping $\overline{a}^\star(\rho)$ and the optimal cell density-MP abundance mapping $\overline{m}^\star(\rho)$ as

$$\overline{m}^\star(\rho) = Z^{-1}\Big(Z_m P(\rho)\Big) \quad \text{and} \quad \overline{a}^\star(\rho) = \left[f_m^{(1)}\right]^{-1}\left(\frac{m_- \exp\Big(Z_a P(\rho)\Big)}{\tau_m(\rho) f_{\text{int},-}}\right), \tag{52}$$

where the superscript $^{-1}$ denotes inverse functions. To explicitly determine the dynamics of the optimal quorum-sensing response, we need to express $\overline{a}^\star$ and $\overline{m}^\star$ as a function of the time over the growth period T. By definition of the input distribution of cell densities, it is easy to see that $P(\rho) = t/T$, where $P(\rho)$ is the cumulative distribution of $p(\rho)$. Thus, the optimal time course of the AI concentration $\overline{a}^\star(t)$ and of the MP abundance $\overline{m}^\star(t)$ can be deduced from a simple substitution $\rho \to P^{-1}\big(t/T\big)$. From the time course $\overline{m}^\star(t)$, we can compute the distribution of MP abundance $q^\star(m)$ in the small-noise approximation, which proves to satisfy $q^\star(m) \propto 1/F^{(1)}(m)$. That is, the optimal output distribution $q^\star(m)$ is inversely proportional the Fano factor of MP expression $F^{(1)}(m)$ in the absence of feedback.

Optimal external and internal feedbacks

Next, we deduce the functional form of the optimal external feedback $f_{\text{ext}}^\star$ and of the optimal internal feedback $f_{\text{int}}^\star$ from the optimal mappings $\overline{a}^\star(\rho)$ and $\overline{m}^\star(\rho)$. As both the feedback functions $f_{\text{ext}}^\star$ and $f_{\text{int}}^\star$ only depend on the MP abundance, we make use of the substitution $\rho \to P^{-1}\big(Z(m)/Z_m\big)$, which is derived from Eqs. (47). Then, together with Eqs. (47) and the self-consistent relations (19), this substitution yields explicit expressions for $f_{\text{ext}}^\star$ and $f_{\text{int}}^\star$:

$$f_{\text{ext}}^\star(m) = f_m^{(1)^{-1}}\left(\frac{m_- \exp\Big(Z_a Z(m)/Z_m\Big)}{\tau_m\big[P^{-1}\left(Z(m)/Z_m\right)\big] f_{\text{int},-}}\right)\Bigg/\Big(\tau_a\, P^{-1}\left(Z(m)/Z_m\right)\Big)\,, \tag{53}$$

$$\frac{f_{\text{int}}^\star(m)}{f_{\text{int},-}} = \frac{m}{m_-}\exp\Big(-Z_a Z(m)/Z_m\Big)\,. \tag{54}$$

Observe that, while the optimal external feedback $f_{\text{ext}}^\star$ depends on the growth regime of the colony through $P(\rho)$ and on the bare output rate $f_m^{(1)}$, the optimal internal feedback $f_{\text{int}}^\star$ depends only on the noise model of MP expression via the Fano factor $F^{(1)}$.

Optimal fluctuations of MP abundance

We can also compute the variance $\big(\Sigma_m^\star\big)^2$ of MP abundance during the optimal quorum-sensing response. From definition (26) and Eq. (46), we deduce that

$$\left(\frac{\overline{m}^{\star\prime}(\rho)}{\Sigma_m^\star(\rho)}\right)^2 = Z_m Z_a\, p(\rho)\,. \tag{55}$$

Using again Eq. (46) to make $\overline{m}^{\star\prime}(\rho)$ explicit and using Eq. (48) to make Z_a and Z_m explicit, we find that

$$\Sigma_m^\star = F^{(1)}(\overline{m})\,(Z_m/Z_a)^{1/2} = F^{(1)}(\overline{m})\left(\int_{m_-}^{m_+}\frac{dm}{F^{(1)}(m)}\Bigg/\ln\left(\frac{m_+ f_{\text{int},-}}{m_- f_{\text{int},+}}\right)\right)^{1/2}\,. \tag{56}$$

As it turns out, such an expression for the optimal MP abundance variance $\left(\Sigma_m^\star\right)^2$ does not guarantee that noise is reduced over the whole range (m_-, m_+) when compared to the variance without feedback $\Sigma_m^2 = \overline{m}F^{(1)}(\overline{m})$. At mean MP abundance $\overline{m}$, the MP fluctuations are reduced by the presence of feedbacks when $\Sigma_m^\star < \Sigma_m$, i.e.:

$$\frac{Z_m}{Z_a} < \frac{\overline{m}}{F^{(1)}(\overline{m})} \quad \text{with} \quad Z_m = \int_{m_-}^{m_+} \frac{dm}{F^{(1)}(m)} \quad \text{and} \quad Z_a = \ln\left(\frac{m_+ f_{\text{int},-}}{m_- f_{\text{int},+}}\right). \tag{57}$$

The above inequality can also be obtained by imposing the internal feedback $f_{\text{int}}^\star$ with analytical expression (54) to be decreasing with the MP abundance m: ${f_{\text{int}}^\star}'(m) < 0$. Indeed, in our mean-field approximation, the shared AI concentration signal is a deterministic input that each cell tracks independently; only the internal feedback can reduce intracellular MP fluctuations. Under what conditions does feedback reduce the MP fluctuations over the whole course of the QS response?" The uniform reduction of MP fluctuations requires $f_{\text{int}}^\star$ to be decreasing over (m_-, m_+), implying that inequality (57) is satisfied for any $\overline{m}$ in (m_-, m_+). Inequality (57) can be rewritten in term of the signal-to-noise ratio of the MP level without feedback, defined as $\text{SNR} = \overline{m}/\Sigma_m^{(1)}$:

$$\text{SNR} = \sqrt{\frac{m}{F^{(1)}(m)}} > \sqrt{\frac{Z_m}{Z_a}}\,. \tag{58}$$

For large burst size b, expressions (33) imply that the SNR at low MP abundance scales as $\sqrt{\overline{m}}$ for TF regulation, while the smaller effective burst size for sRNA allows the SNR to remain high at low MP abundance. Thus, for small enough value of m_-, inequality (58) can fail at low MP abundance for TF regulation, but hold at all MP abundances for sRNA regulation. As a result, for TF regulation, $f_{\text{int}}^\star$ can be increasing at low MP abundance and amplify MP fluctuations, whereas, for sRNA regulation, $f_{\text{int}}^\star$ remains decreasing over the whole range (m_-, m_+), thereby reducing MP fluctuations over the full range of the quorum-sensing response.

3.2 Optimal quorum-sensing response

In order to fully characterize the optimal quorum-sensing response, we need to specify the functional form for the bare output rate $f_m^{(1)}$ describing the upregulation of MP expression by AI detection. For simplicity, we consider that $f_m^{(1)}$ follows a Hill activation curve, i.e.

$$f_m^{(1)}(a) = \alpha + \beta \frac{a^h}{K^h + a^h}\,. \tag{59}$$

with induction constant K and Hill coefficient h [9]. The MP gene is expressed at a basal rate α when no AI molecules are detected, and at a rate $\alpha + \beta$ for saturating AI concentrations $a \gg K$. Both α and β are determined from the model parameters by the two boundary conditions $\tau_m(\rho_-) f_m^{(1)}(a_-) f_{\text{int},-} = m_-$ and $\tau_m(\rho_+) f_m^{(1)}(a_+) f_{\text{int},+} = m_+$.

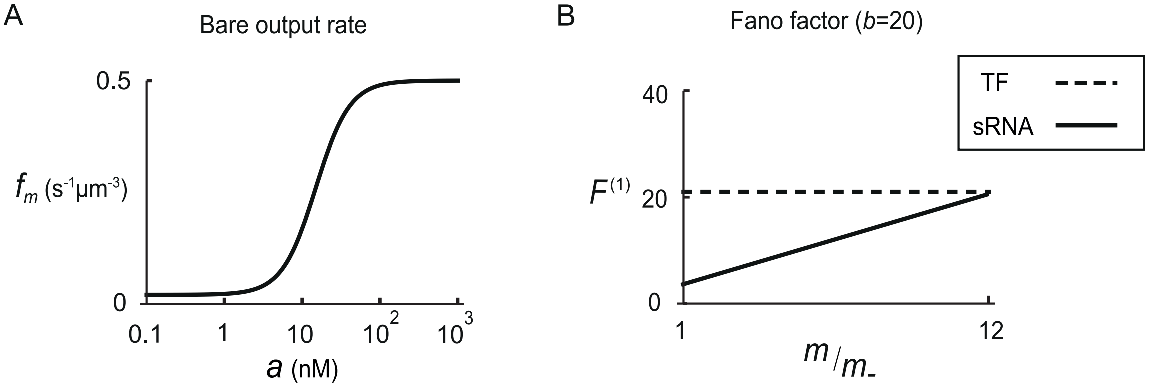

S1 Fig. Models for the regulation of monitor protein expression. In the expressed regime of MPs and for strong sRNA-mRNA pairing: **A.** The mean MP expression rate follows the same Hill function for both TF regulation and sRNA regulation with Hill coefficient $h = 2$, with induction constant $K = 15$nM, and over the range of AI concentration (a_-, a_+) with $a_- = 0.1$nM and $a_+ = 1\mu$M. **B.** sRNA regulation reduces the stochasticity in MP expression compared with TF regulation, as revealed by the scaling of the Fano factors $F = \Sigma_m^2/\overline{m}$ where, for simplicity, we take the saturation level of MP abundance to be $m_\infty = \tau_m(\alpha + \beta) \approx m_+/f_{\text{int},+} = 1200$nM with $m_- = 100$nM and $m_+ = 600$nM.

Then, considering the two simple models of MP expression of Section 2.2, TF regulation and sRNA regulation, allows us to specify functional forms for the Fano factor $F^{(1)}$ using Eqs. (33) and (34). Together with the bare output rate $f_m^{(1)}(a)$, the Fano factor $F^{(1)}(m)$ characterizes the noisy regulation of MP expression, which fundamentally limits the fidelity of the quorum-sensing channel. We represent our choice of $f_m^{(1)}$ and $F^{(1)}(m)$ in S1 Fig. To specify $p(\rho)$ the input distribution of cell densities, we consider the regimes of bacterial growth prescribed by (7) and parametrized by a single exponent γ. Recall that for $\gamma = 0$, the colony grows exponentially and that we only consider $\gamma \approx 0$ (see S2 Fig A). In any case, we have $p(\rho) \propto \rho^{\gamma-1}$ and we see that the larger γ, the larger the fraction of time that bacteria spend at high cell density.

Role of feedbacks

In S2 Fig, we represent the optimal quorum-sensing response for our choice of $f_m^{(1)}$ and $F^{(1)}(m)$ in the case of a constant MP lifetime τ_m. With $\mu = 1$, a constant τ_m corresponds to exponential growth. For general growth, if the MPs are only limited by dilution, a constant τ_m is ensured by taking the metabolic coefficient proportional to the growth rate: $\mu(\rho) \propto 1/\tau_d(\rho)$. For such instances of our model, we can readily characterize the role of feedbacks in shaping the quorum-sensing response. By our definition, a feedback is positive when it speeds up the transition to high MP abundance in response to increasing

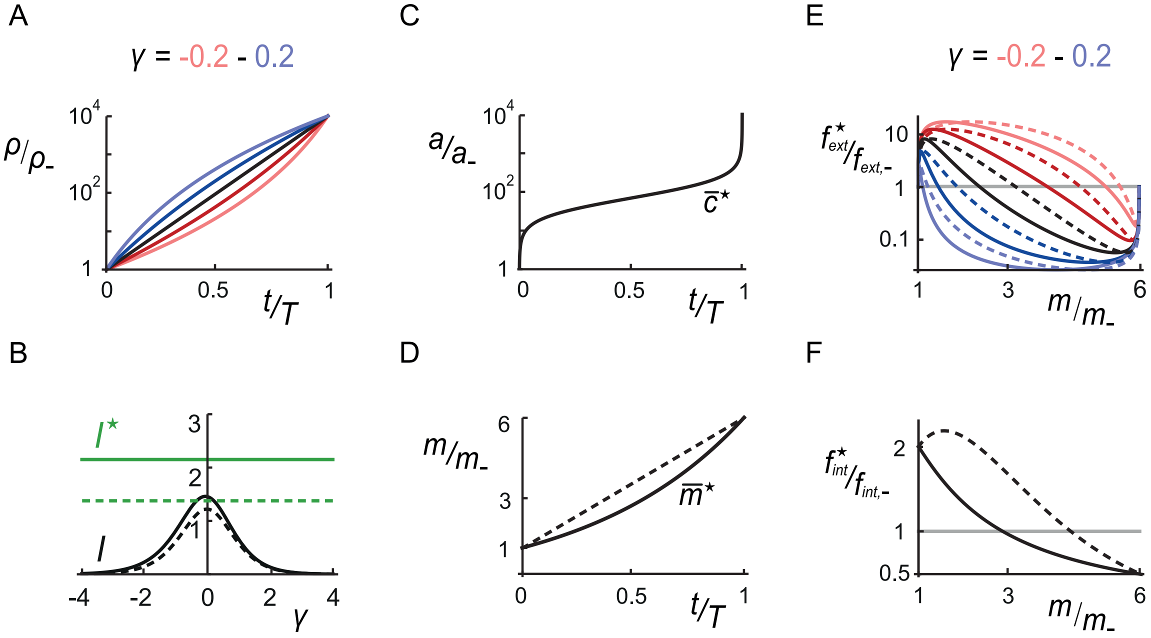

S2 Fig. Optimization of quorum-sensing feedbacks in the mean-field regime. Parameter values: $a_+/a_- = \rho_+/\rho_- = 10^4$, $m_- = 100$nM, $m_+ = 600$nM, $f_{\mathrm{int},-} = 2$, $f_{\mathrm{int},+} = 1/2$, $K/a_- = 150$, $h = 2$, $b = 20$. **A.** Temporal dynamics of the cell density growth for different growth exponents $\gamma = -0.2, -0.1, 0, 0.1, 0.2$. **B.** Optimal MI $I^\star$ with feedback (green curve) and MI I without feedback (black curves), both in bits, as functions of the growth exponent γ, for TF regulation (dashed curves) and sRNA regulation (solid curves). **C.** Optimal time course of the AI concentration. **D.** Optimal time course of the MP abundance. **E.** Optimal external feedback $f^\star_{\mathrm{ext}}$. **F.** Optimal internal feedback $f^\star_{\mathrm{int}}$. In **E.** and **F.** the gray lines indicate the mean response in the absence of feedback ($f'_{\mathrm{ext}} = 0, f_{\mathrm{int}} = 1$). For constant lifetime τ_m, only the optimal external feedback represented in **E** depends on the growth exponent γ. In all panels, the dashed curves correspond to TF regulation and the solid curves to sRNA regulation.

cell density, i.e. when a feedback function is an increasing function of MP abundance m. Similarly, a feedback is negative when it slows down the transition to high MP abundance in response to increasing cell density, i.e. when a feedback function is a decreasing function of MP abundance m. In particular, the feedbacks $f^\star_{\mathrm{ext}}$ and $f^\star_{\mathrm{int}}$ can switch between positive and negative over the range (m_-, m_+). Equipped with these definitions, we can distinguish the role of the external feedback f_{ext} (the AI output rate normalized by cell volume) from the role of the internal feedback f_{int} (the self-regulation function).

External feedback: Observing for our choice of $f^{(1)}_m(a)$ in S2 Fig, the detection channel has low resolution power for AI concentrations that are significantly different from the induction constant K, where it has maximum resolution. Given some boundary conditions on a_- and a_+, the effect of external feedback is to maximize the fraction of time that the detection channel operates at high resolution as shown in S2 Fig C and S2 Fig D. If $a_- \ll K$ ($K \ll a_+$), the initial (final) positive feedback allows the quorum-sensing system to skip AI concentrations where the MP expression is nearly at base level (at saturation). The negative feedback ensures that the AI concentration spends most of its time in the range of optimal sensitivity, i.e. around K. As opposed to the optimal time course of AI concentration $\overline{a}(t)$, the optimal external feedback $f^\star_{\mathrm{ext}}$ depends on the growth regime. Actually, the magnitude of positive (negative) feedback increases with slower (faster) growth to high cell density, i.e. with decreasing (increasing) growth exponent γ.

Internal feedback: Assuming self-induction at low MP expression ($f_{\mathrm{int},-} = 2 > 1$) and self-repression at high MP expression ($f_{\mathrm{int},+} = 1/2 < 1$), the optimal internal feedback $f^\star_{\mathrm{int}}$, as an overall decreasing function, reduces the fluctuations of MP abundance. For a fixed bare output rate $f^{(1)}_m$, the optimal internal feedback $f^\star_{\mathrm{int}}$ (see S2 Fig D) only depends on the bare Fano factor $F^{(1)}$: For sRNA regulation, the optimal feedback is graded, controlling MP fluctuations evenly throughout the range of MP variation. For TF regulation, by contrast, the optimal feedback is positive in the low expression regime, where the resolution of the detection channel is particularly low. In this case, it is beneficial to exploit feedback to skip low MP abundance, thereby transiently amplifying fluctuations, in order to implement a strong negative feedback for higher MP levels. For both models of regulation, the effect of the optimal feedback $f^\star_{\mathrm{int}}$ is to extend the range of MP abundance over which the detection channel has good resolving power. In fact, the optimal time course of MP abundance is exactly linear for TF regulation and nearly linear for sRNA regulation, as shown in S2 Fig C.

We can now investigate the contributions of the optimal feedbacks to improving the information transfer of quorum sensing. For conciseness, we only give a brief account of these contributions based on S2 Fig B, which represents the optimal MI $I^\star_{\rho,m}$ and the MI $I^{(1)}_{\rho,m}$ without feedback for various regimes of growth. As apparent, for both regulation

models, the MI $I^{(1)}_{\rho,m}$ is maximal for the regime of exponential growth ($\gamma = 0$). In fact, one can check that the MI $I^{(1)}_{\rho,m}$ for $\gamma = 0$ is close to the capacity of the detection channel in the absence of internal feedback ($f_{\text{int}} = 1$). For the boundary conditions we consider on $f_{\text{int},-}$ and $f_{\text{int},+}$, the external feedback only substantially contributes to information transfer when bacterial growth noticeably deviates from the exponential regime. We thus deduce that, when feedbacks produce a substantial information increase at exponential growth, most of that increase is due to the negative internal feedback.

Validity of the mean-field regime

Prior to deriving the analytical formulae for the optimal feedbacks $f^\star_{\text{ext}}$ and $f^\star_{\text{int}}$, we made a series of biologically relevant approximations to model the quorum-sensing response in the mean-field regime (see Section 2.1). Specifically, we made the assumption that quorum-sensing bacteria act as independent sensors operating in a regime where both the small-noise and quasi-static approximations, which may not be justified for any instance of feedbacks. Here, we validate *a posteriori* these approximations for the optimal quorum-sensing system.

(i) In principle, positive external feedback correlates quorum sensing between bacteria and might cause our approximations to fail. Fortunately, the optimal quorum-sensing response maximizes the fraction of time AI detection is performed with high resolution via negative feedback, i.e. the fraction of time when the external feedback function $f^\star_{\text{ext}}$ is decreasing. For decreasing functions $f^\star_{\text{ext}}$, expressions (21) and (22) show that the covariance of MP abundances between bacteria satisfies $C_m \leq \Sigma^2_m/N$, where Σ^2_m is the MP variance and N is the number of bacteria in the colony, thereby justifying the treatment of MP fluctuations as independent. Thus, when the optimal quorum-sensing system processes information in large colonies ($N > 100$) and when the external feedback function $f^\star_{\text{ext}}$ is decreasing, bacteria act as independent detectors of the mean-field AI concentration.

(ii) The quasi-static and small-noise approximation are central to the formulation of the quorum-sensing information channel. However, biological considerations suggest that (1) the quorum-sensing timescale τ_m and the timescale of growth τ_d are of the same order and that (2) the high abundance of MPs implies large burst sizes ($b = 20$), with possibly substantial noise. To validate the quasi-static and small-noise approximations, we numerically simulated the optimal response of the detection channel for bacteria independently measuring the AI concentration signal $\overline{a}(t)$. Using the analytical expressions for the optimal time course $\overline{a}^\star$ and the optimal internal feedback $f^\star_{\text{int}}$, the MP fluctuation in a cell is described via the stochastic differential equation:

$$dm_t = \frac{m_t}{\tau_m}\left(-1 + \left(\frac{m_+ f_{\text{int},-}}{m_- f_{\text{int},+}}\right)^{\frac{t}{T} - \frac{Z(m)}{Z_m}}\right) dt + \sqrt{2}\sigma_m \, dW_t \,. \tag{60}$$

As shown in S3 Fig, the quasi-static and small-noise approximations hold well for sRNA

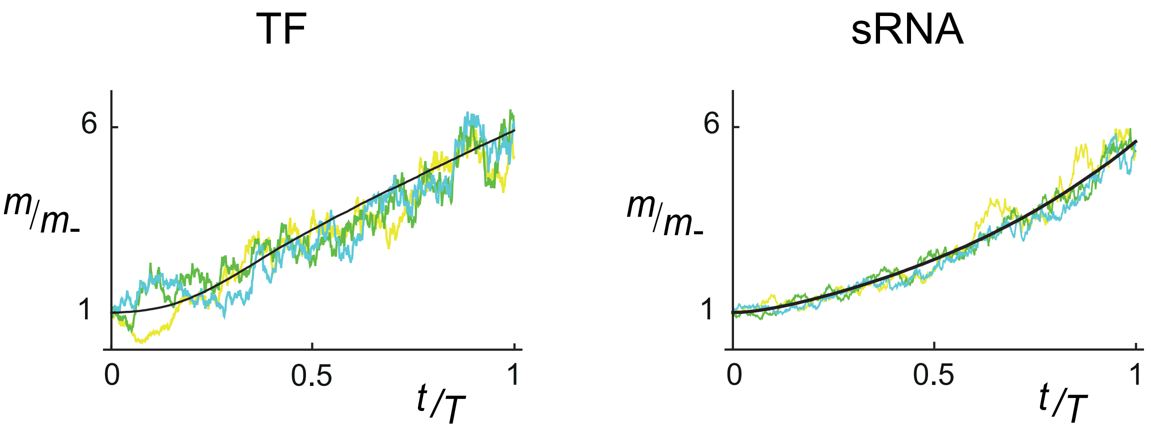

S3 Fig. Numerical simulation of the optimal quorum-sensing response. Time course of the MP abundance in response to the optimal time course of AI concentration and with optimal internal feedback: the colored curves represent three independent realizations of the time course and the black line represents the empirical mean time course.

regulation, and for TF regulation, albeit to a lesser degree. Indeed, S3 Fig shows that random realizations of the time courses $a(t)$ and $m(t)$ closely follow their respective empirical means and the analytical curves in S2 Fig C. The validity of these approximations is in large part due to the optimal internal feedback $f^{\star}_{\mathrm{int}}$ which is negative over the range of MP abundance where information processing takes place. Indeed, the effects of a negative internal feedback f_{int} are (1) to shorten the effective MP lifetime, thus slaving the MP abundance m to the slower variable ρ, and (2) to dampen the MP abundance fluctuations, thus controlling noise in the response.

Numerical approximations and caveats

The above considerations validate the functional forms of $f^{\star}_{\mathrm{ext}}$ and $f^{\star}_{\mathrm{int}}$ as optimal feedbacks for our quorum-sensing model in the small-noise approximation. However, quorum-sensing systems appear to operate with significant burst sizes [10] for which the small-noise approximation may fail on two accounts: First, the feedbacks $f^{\star}_{\mathrm{ext}}$ and $f^{\star}_{\mathrm{int}}$, obtained by variational optimization of the small-noise MI $\tilde{I}_{\rho,m}$ (41), increasingly fail to be optimal for large burst sizes. Second, even if the optimal feedback remains valid for large burst sizes, the approximate MI $\tilde{I}^{\star}_{\rho,m}$ can still substantially underestimate the real MI $I^{\star}_{\rho,m}$. In this subsection, we discuss the implications of these two caveats.

Before discussing the caveats of the small-noise approximation, we note that, for exponential growth, the optimal internal feedback $f^{\star}_{\mathrm{int}}$ is responsible for most of the information

benefit. Focusing on $f^\star_{\rm int}$ rather than $f^\star_{\rm ext}$, we rewrite expression (54) as

$$\frac{f^\star_{\rm int}(m)}{f_{{\rm int},-}} = \frac{m}{m_-}\left(\frac{m_- f_{{\rm int},+}}{m_+ f_{{\rm int},-}}\right)^{\frac{\int_{m_-}^{m} 1/F^{(1)}(u)\,du}{\int_{m_-}^{m_+} 1/F^{(1)}(u)\,du}}, \tag{61}$$

to stress the dependence of the functional form of $f^\star_{\rm int}$ on the ranges (m_-, m_+), $(f_{{\rm int},-}, f_{{\rm int},+})$, and on the MP Fano noise function $F^{(1)}(m)$. To clarify the discussion, we distinguish between the role of the boundary conditions m_-, m_+, $f_{{\rm int},-}$, $f_{{\rm int},+}$, and the role of the Fano function $F^{(1)}(m)$, which depends on the burst size b: For fixed MP range (m_-, m_+), self-induction at low MP abundance $f_{{\rm int},-} > 1$ and self-inhibition at high MP abundance $f_{{\rm int},+} < 1$ provides the quorum-sensing system with a "budget" of negative feedback, allowing $f^\star_{\rm int}$ to be decreasing over the range (m_-, m_+). The Fano noise function $F^{(1)}(m)$ specifies the signal-to-noise ratio of the MP level without feedback SNR $= \overline{m}/\Sigma^{(1)}_m = \sqrt{\overline{m}F^{(1)}(\overline{m})}$, which explicitly reads

$$\text{TF}: \quad \text{SNR} = \sqrt{\frac{\tau_m\langle v\rangle}{1+b}\,\overline{m}}\,, \qquad \text{sRNA}: \quad \text{SNR} = \sqrt{\frac{\tau_m\langle v\rangle}{1+b\overline{m}/m_\infty}\,\overline{m}}\,. \tag{62}$$

Suppose that we have a zero budget of negative feedback ($f_{{\rm int},-} = f_{{\rm int},+} = 1$) for small burst sizes b, so that the small-noise approximation applies. For both TF and sRNA regulation, the SNR is comparable, being worse at low MP abundance than at high MP abundance. S4 Fig A shows that, as a result of the variational optimization of the small-noise MI, optimal feedbacks for both regulations are first positive (increasing $f^\star_{\rm int}$ at low MP abundance), then negative (decreasing $f^\star_{\rm int}$ at high MP abundance). While positive feedback amplifies fluctuations at low MP abundance, the net level of self-induction $f^\star_{\rm int} > f_{{\rm int},-} = 1$ at mid MP level makes it possible for the feedback to be negative at high MP abundance, thereby reducing fluctuations for MP levels with best SNR. This trade-off between positive and negative feedback produces a substantial overall information gain for small burst size (0.5 bits for $b = 1$).

For larger burst sizes b, the TF SNR is worse over the whole MP range, whereas the sRNA SNR primarily worsens at large MP abundance, due to the shorter effective lifetime of MP mRNA at low MP abundance ($b\overline{m}/m_\infty < b$). Then, for sRNA regulation, trading-off positive feedback at low MP abundance for negative feedback at high MP abundance becomes less advantageous: S4 Fig A shows that the optimal feedback $f^\star_{\rm int}$ becomes flat for $b = 20$ and tuning the internal feedback offers minimal information gain for sRNA regulation with $f_{{\rm int},-} = f_{{\rm int},+} = 1$. By contrast, the optimal feedback for TF regulation is unchanged and may provide the quorum-sensing system with an information benefit. However, such an optimal feedback $f^\star_{\rm int}$ has been obtained by optimizing the small-noise MI, which becomes a poor approximation for the real MI for large burst size. We thus expect the information benefit of our optimal internal feedback to vanish for large enough

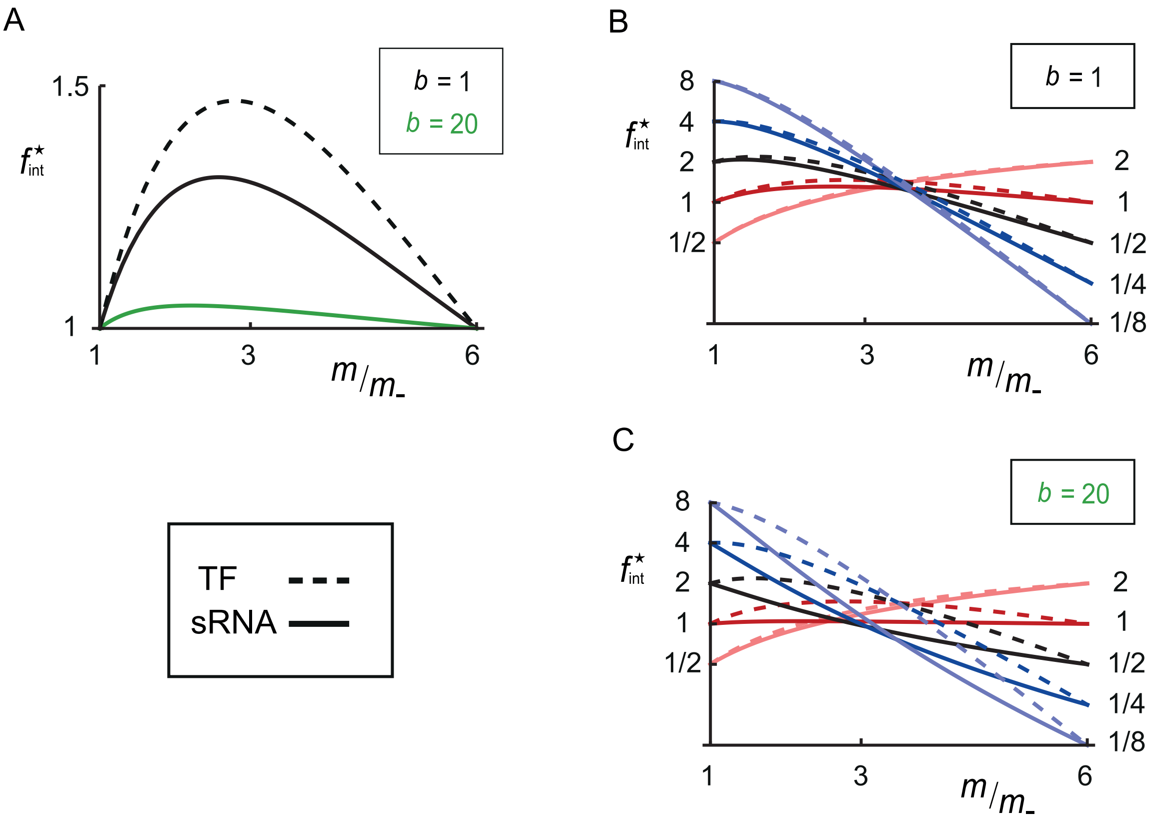

S4 Fig. Optimal internal feedbacks. A. For zero budget of negative feedback with small burst size ($f_{\text{int},-} = f_{\text{int},+} = 1, b = 1$), $f^\star_{\text{int}}$ is increasing then decreasing for both TF and sRNA regulations. For large burst sizes, $f^\star_{\text{int}}$ is unchanged for TF regulation but becomes nearly flat for sRNA regulation. **B.** For small burst size, a finite budget of negative feedback ($f_{\text{int},-} > 1, f_{\text{int},+} < 1, b = 1$) allows $f^\star_{\text{int}}$ to be decreasing over the whole MP range. **C.** For large burst sizes ($b = 20$), the optimal feedback $f^\star_{\text{int}}$ expends its budget of negative feedback preferentially at low MP abundance for sRNA regulation, as opposed to the TF regulation case. The level of self regulation $f^\star_{\text{int}}$ is represented in linear scale in **A** and in logarithmic scale in **B** and **C**.

burst sizes b. For TF regulation with $f_{\text{int},-} = f_{\text{int},+} = 1$, we actually find no information gain via feedback for $b > 10$.

Now, suppose that we have a nonzero budget for negative feedback with self-induction at low MP abundance and self-inhibition at high MP abundance, e.g. $f_{\text{int},-} = 2$ and $f_{\text{int},-} = 1/2$. The internal feedback $f^{\star}_{\text{int}}$ can then control noise by expending its budget of negative feedback, without trading it off for an initial positive feedback. For small burst size, S4 Fig B shows that, for both TF and sRNA regulations, the optimal feedback is decreasing over the whole MP range (m_-, m_+) for large level of self-regulation ($f_{\text{int},-} > 2, f_{\text{int},+} < 1/2$). For large burst size, S4 Fig C shows that the optimal feedbacks for sRNA regulation substantially differ from the unchanged optimal feedbacks for TF regulation. As for the case $f_{\text{int},-} = f_{\text{int},+} = 1$, large burst sizes primarily deteriorate the sRNA SNR at large MP abundance. Thus, for larger burst sizes b, controlling noise via negative feedback at large MP abundance becomes less advantageous compared with controlling noise at low MP abundance. S4 Fig C reveals that, for sRNA regulation and by contrast with TF regulation, the optimal quorum-sensing response spends most of its budget of negative feedback at low MP concentration ($b = 20$). As opposed to the case $f_{\text{int},+} = f_{\text{int},-} = 1$, we find that our optimal feedbacks, obtained by optimizing the small-noise MI, yield an information benefit even for large burst sizes.

In fact, the optimal MI $\tilde{I}^{\star}_{\rho,m}$, as a small-noise approximation, is only a lower bound to the true MI $I^{\star}_{\rho,m}$ of the quorum-sensing channel with optimal feedbacks $f^{\star}_{\text{ext}}$ and $f^{\star}_{\text{int}}$. However, we can still use $f^{\star}_{\text{ext}}$ and $f^{\star}_{\text{int}}$ to more accurately numerically compute the MI $I^{\star}_{\rho,m}$ in the mean-field regime. For large burst sizes $b \geq 10$, it turns out that the small-noise MI $\tilde{I}^{\star}_{\rho,m}$ consistently underestimates $I^{\star}_{\rho,m}$. This error results from defining the output distribution of MP abundance $q(m)$ as the deterministic transform of $p(\rho)$ through the cell density-MP abundance mapping $\overline{m}(\rho)$. Such a definition neglects the spreading of $q(m)$ due to the stochasticity in MP abundance, thus underestimating the entropy of $q(m)$. In particular, the use of the MI $\tilde{I}^{\star}_{\rho,m}$ assumes that $q(m)$ is strictly defined over (m_-, m_+), an approximation that becomes worse for larger burst size b. To accurately report the MI, we chose to plot the numerical estimates of the true MI $I^{\star}_{\rho,m}$ associated with the optimal feedbacks $f^{\star}_{\text{ext}}$ and $f^{\star}_{\text{int}}$ in S2 Fig B. To validate the near optimality of the feedbacks, we can then use the Blahut-Arimoto algorithm to compute numerically the exact capacity $C^{\star}_{\rho,m}$ associated with $f^{\star}_{\text{ext}}$ and $f^{\star}_{\text{int}}$, without making the small-noise approximation [14]. For an information channel with a continuous variable, the exact capacity is generally reached for singular input distributions, which are not physically realistic and lead to strong numerical discontinuities [15]. When evaluated numerically, the physically realistic MI $I^{\star}_{\rho,m}$ captures more than 80% of the exact capacity $C^{\star}_{\rho,m}$ (see S5 Fig).

3.3 Optimal capacity of quorum sensing

In Section 3.1, we optimize the quorum-sensing MI $\tilde{I}_{\rho,m}$ by varying the encoding scheme $p\big(\{m_i\}|\rho\big)$ at fixed input distribution $p(\rho)$. We can also maximize the MI $\tilde{I}_{\rho,m}$ by varying

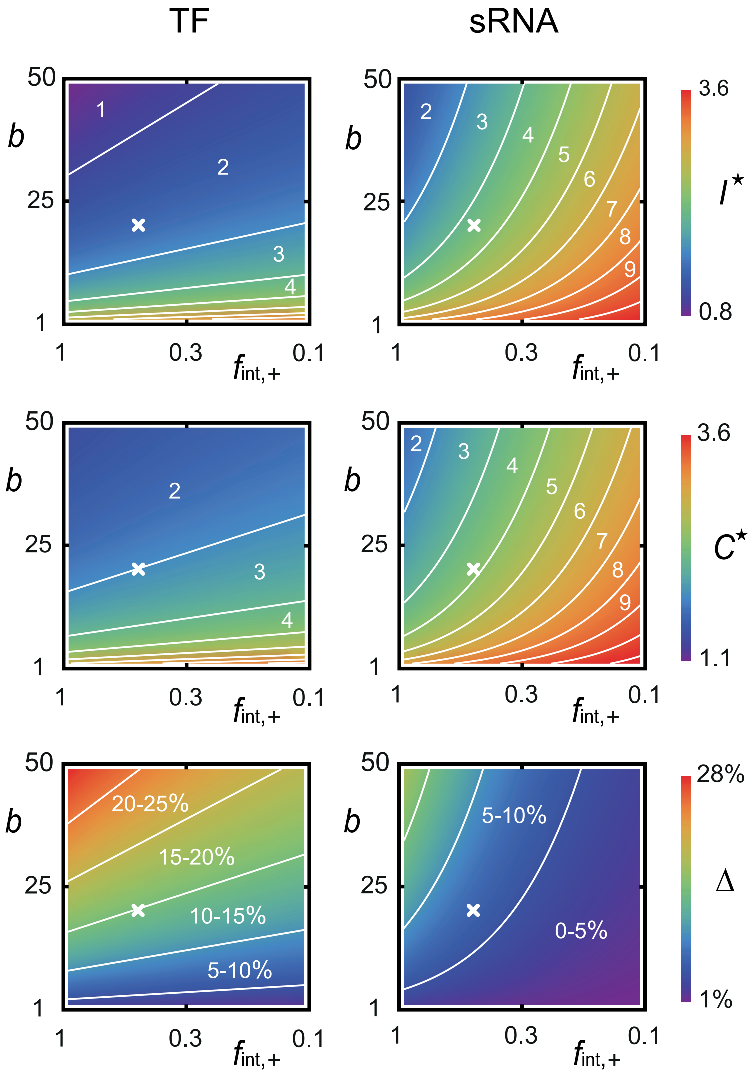

S5 Fig. Capacity and numerical MI. Dependence of the optimal MI $I^\star$, the exact capacity $C^\star$ and the MI relative error Δ on the burst size b and on the level of self-repression $f_{\mathrm{int},+}$ (in logarithmic scale) for both TF and sRNA regulations. The Xs indicate the values $f_{\mathrm{int},+} = 1/2$ and $b = 20$ for which Table 1 was computed. The top panels reproduce the numerical MI values plotted in Fig. 5 of the main manuscript. The middle panels shows the exact capacity computed via the Blahut Arimoto algorithm and using the analytically obtained feedbacks. In the top and middle panels, the white curves are isoinformation curves separating regions where the optimal quorum-sensing channel can discriminate the indicated number of cell-density ranges. Parameter values: $\rho_+/\rho_- = 10^4$, $a_- = 0.1$nM, $a_+ = 1$mM, $m_- = 100$nM, $m_+ = 600$nM, $f_{\mathrm{int},-} = 2$, $K = 15$nM, $h = 2$ and $v = 1\mu\mathrm{m}^3$.

the input distribution $p(\rho)$ at fixed encoding scheme $p\big(\{m_i\}|\rho\big)$. This optimization yields the small-noise capacity $\tilde{C}_{\rho,m}$, i.e. the maximal amount of information transmitted by a given quorum-sensing channel. In this section, we maximize the small-noise capacity $\tilde{C}_{\rho,m}$ by varying the feedback functions f_{ext} and f_{int} and show that the optimal capacity $\tilde{C}^{\star}_{\rho,m}$ actually coincides with the optimal MI $\tilde{I}^{\star}_{\rho,m}$.

Capacity optimization with variable MP lifetime

In the small-noise regime, the integral expression for the capacity $\tilde{C}_{\rho,m}$ of the quorum-sensing information channel is given by

$$\tilde{C}_{\rho,m} = \log_2\left(\int_{\rho_-}^{\rho_+} \frac{d\rho}{\sqrt{2\pi e}\,\delta_\rho}\right)\,, \tag{63}$$

where $\delta_\rho = \Sigma_m(\rho)/\overline{m}'(\rho)$ is the smallest difference in MP abundance that a bacterium can resolve by reading out its MP abundance [15]. The capacity $\tilde{C}_{\rho,m}$ can be written as a functional of the cell density-AI concentration mapping $\overline{a}(\rho)$ and the cell density-MP abundance mapping $\overline{m}(\rho)$

$$\tilde{C}_{\rho,m}\big[\overline{m},\overline{a}\big] = \log_2\left(\frac{1}{\sqrt{2\pi e}}\int_{\rho_-}^{\rho_+} \mathcal{L}\big[\overline{a}'(\rho),\overline{a}(\rho),\overline{m}'(\rho),\overline{m}(\rho),\rho\big]\,d\rho\right)\,, \tag{64}$$

where the Lagrangian $\mathcal{L}$ has the form

$$\mathcal{L}[\overline{a}',\overline{a},\overline{m}',\overline{m},\rho] = \sqrt{\frac{\overline{m}'}{F^{(1)}(\overline{m})}}\sqrt{\frac{\partial_\rho\big[\tau_m(\rho)f_m^{(1)}(\overline{a})\big]}{\tau_m(\rho)f_m^{(1)}(\overline{a})}}\,. \tag{65}$$

Optimizing the capacity $\tilde{C}_{\rho,m}$ over $\overline{a}$ and $\overline{m}$, i.e. over the external feedback f_{ext} and the internal feedback f_{int}, is a problem in the calculus of variations. By the same mathematical arguments as in Section 3.1, we can show that, if a stationary path $(\overline{a}^\star,\overline{m}^\star)$ solves the associated Euler-Lagrange equations, it is the optimal path, i.e. the path that maximizes the capacity $\tilde{C}_{\rho,m}$. The Euler-Lagrange equation corresponding to varying the cell density-AI concentration mapping $\overline{a}(\rho)$ is

$$\frac{\partial\mathcal{L}}{\partial\overline{a}} - \frac{d}{d\rho}\left(\frac{\partial\mathcal{L}}{\partial\overline{a}'}\right) = 0\,. \tag{66}$$

This equation can be integrated to yield the first integral

$$\frac{\partial_\rho\big[\tau_m f_m^{(1)}(\overline{a})\big]}{\tau_m f_m^{(1)}(\overline{a})} = \frac{Z_a}{Z_m}\frac{\overline{m}'}{F^{(1)}(\overline{m})}\,, \tag{67}$$

where Z_a and Z_m are defined as in (48). To determine the constant Z_a/Z_m, we have used the boundary conditions $\overline{a}(\rho_-) = a_-$, $\overline{a}(\rho_+) = a_+$ and $\overline{m}(\rho_-) = m_-$, $\overline{m}(\rho_+) = m_+$ in the integral version of the above first integral:

$$\tau_m f_m^{(1)}(\overline{a}) = (m_-/f_{\text{int},-}) \exp\left((Z_a/Z_m) Z(\overline{m})\right). \tag{68}$$

Together with the self-consistent relations (19), the relation (68) completely determines the quorum-sensing response with optimal capacity. Thus, as opposed to the case of the MI $\tilde{I}_{\rho,m}$, the optimization of the capacity $\tilde{C}_{\rho,m}$ is done in a single step, since the channel input distribution is already optimized at capacity. The cell density-AI concentration mapping and cell density-MP abundance mapping corresponding to optimal capacity prove to be $\overline{a}^\star(\rho)$ and $\overline{m}^\star(\rho)$. The optimal capacity is then identical to the optimal MI

$$\tilde{C}^\star_{\rho,m} = \tilde{C}_{\rho,m}\left[\overline{a}^\star, \overline{m}^\star\right] = \frac{1}{2}\log_2\left(\frac{Z_a Z_m}{2\pi e}\right) = \tilde{I}^\star_{\rho,m}. \tag{69}$$

Thus, the optimal MI $\tilde{I}^\star_{\rho,m}$ at fixed input distribution $p(\rho)$ achieves the theoretical optimal information transfer, i.e the optimal capacity $\tilde{C}^\star_{\rho,m}$, of our quorum-sensing models.

Capacity optimization with constant MP lifetime

If the growth function is exponential, i.e. τ_d is a constant, then the MP lifetime τ_m is constant and the quorum-sensing information channel has the structure $\rho \to a \leftrightarrows m \circlearrowleft$. As the internal MP abundance m directly depends on τ_m, the general quorum-sensing channel has an additional arrow going from ρ to m. We recall that, for general growth, if the MPs are only limited by dilution, a constant τ_m is ensured by taking the metabolic coefficient proportional to the growth rate: $\mu(\rho) \propto 1/\tau_d(\rho)$. Then, in the mean-field regime of quorum sensing, one can arbitrarily shape the distribution of AI concentration $q(a)$ by varying the self-averaging external feedback f_{ext}. This suggests that one should consider the AI concentration a as the free input of the truncated detection channel $a \to m \circlearrowleft$. S6 Fig summarizes this approach schematically. The detection channel $a \to m \circlearrowleft$ only depends on the internal quorum-sensing circuit, i.e. on the bare output rate $f_m^{(1)}$, the bare Fano factor $F^{(1)}$, and the internal feedback f_{int}. The small-noise capacity of the detection channel is given by

$$\tilde{C}_{a,m} = \log_2\left(\int_{a_-}^{a_+} \frac{da}{\sqrt{2\pi e}\,\delta_a}\right), \tag{70}$$

where $\delta_a = \Sigma_m(a)/\overline{m}'(a)$ is the smallest difference in AI concentration that a bacterium can resolve by reading out its MP abundance. The capacity $\tilde{C}_{a,m}$ can be written as a functional of the AI concentration-MP abundance mapping $\overline{m}(a)$

$$\tilde{C}_{a,m}\left[\overline{m}\right] = \log_2\left(\frac{1}{\sqrt{2\pi e}}\int_{\rho_-}^{\rho_+} \mathcal{L}\left[\overline{m}'(a), \overline{m}(a), a\right] da\right), \tag{71}$$

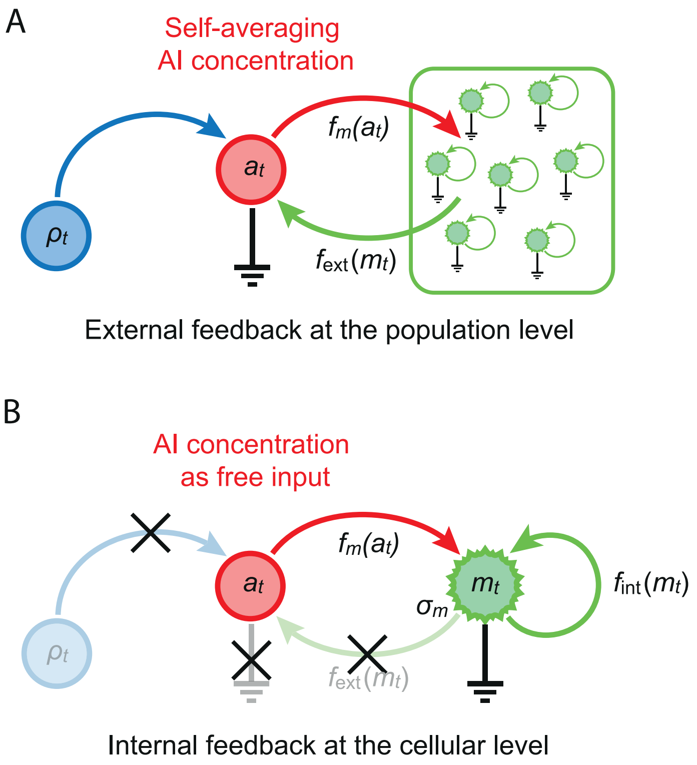

S6 Fig. Optimal MI as capacity. A. The fluctuations of the AI concentration self-average over the slow timescale $\tau_m \geq 30$min and the large population of bacteria $N \geq 100$. **B.** Varying f_{ext} allows us to arbitrarily modify the distribution of the AI concentration a, which can be seen as a free input. As a result, the optimal information transfer of the quorum-sensing channel is the optimal information capacity of the embedded detection channel.

where the Lagrangian $\mathcal{L}$ has the form

$$\mathcal{L}[\overline{m}', \overline{m}, a] = \left(\frac{\overline{m}'}{F^{(1)}(\overline{m})} \frac{{f_m^{(1)}}'(a)}{f_m^{(1)}(a)} \right)^{1/2} . \tag{72}$$

Optimizing the capacity $\tilde{C}_{a,m}$ over $\overline{m}$, i.e. over the internal feedback f_{int}, is a problem in the calculus of variations. Again, by the same mathematical arguments as in Section 3.1, we can show that, if a stationary path $\overline{m}^\star$ solves the Euler-Lagrange equation

$$\frac{\partial \mathcal{L}}{\partial \overline{m}} - \frac{d}{d\rho} \left(\frac{\partial \mathcal{L}}{\partial \overline{m}'} \right) = 0 \,, \tag{73}$$

it is the optimal path, i.e. the path that maximizes the capacity $\tilde{C}_{a,m}$. As usual, the Euler-Lagrange equation can be integrated to yield

$$\frac{\overline{m}'}{F^{(1)}(\overline{m})} = \frac{Z_m}{Z_a} \frac{{f_m^{(1)}}'}{f_m^{(1)}} \,, \tag{74}$$

where we have used the boundary conditions $\overline{m}(a_-) = m_-$ and $\overline{m}(a_+) = m_+$ to determine the constant Z_m/Z_a. From there, we deduce the optimal capacity

$$\tilde{C}^\star_{a,m} = \tilde{C}_{a,m}\left[\overline{m}^\star\right] = \log_2 \left(\sqrt{\frac{Z_m/Z_a}{2\pi e}} \int_{a_-}^{a_+} \frac{{f_m^{(1)}}'(a)}{f_m^{(1)}(a)} \, da \right) = \tilde{I}^\star_{\rho,m} \,, \tag{75}$$

which establishes that, for exponential growth or when the metabolic coefficient is proportional to the rate of growth, the optimal MI $I^\star_{\rho,m}$ of $\rho \to a \leftrightarrows m \circlearrowleft$ is equal to the optimal capacity $\tilde{C}^\star_{a,m}$ of the detection channel $a \to m \circlearrowleft$. Moreover, when there is no self-regulation in the low and high MP expression regime, i.e. when $f_{\text{int},-} = f_{\text{int},+} = 1$, we can verify that the optimal MI $\tilde{I}^\star_{\rho,m}$ is larger than the capacity of the detection channel in the absence of internal feedback

$$\tilde{C}^{(1)}_{\rho,m} = \log_2 \left(\frac{1}{\sqrt{2\pi e}} \int_{m_-}^{m_+} \frac{dm}{\sqrt{m F^{(1)}(m)}} \right) . \tag{76}$$

This is a consequence of the Cauchy-Schwarz inequality applied to the functions $m \mapsto 1/\sqrt{m}$ and $m \mapsto 1/\sqrt{F^{(1)}(m)}$ in the space $L^2(m_-, m_+)$:

$$\int_{m_-}^{m_+} \frac{dm}{\sqrt{m F^{(1)}(m)}} \leq \left(\ln\left(\frac{m_+}{m_-}\right) \int_{m_-}^{m_+} \frac{dm}{F^{(1)}(m)} \right)^{1/2} . \tag{77}$$

The above inequality becomes an equality when the functions $m \mapsto 1/\sqrt{m}$ and $m \mapsto 1/\sqrt{F^{(1)}(m)}$ are proportional. Thus, there is no gain of information when $F^{(1)}(m) \propto m$, corresponding to the case of sRNA regulation with large burst size b. In this case, the information gain due to internal feedback follows exclusively from the boundary conditions $f_{\text{int},-} > 1$ and $f_{\text{int},+} < 1$.

References

[1] P. S. Stewart, "Diffusion in biofilms," *Journal of Bacteriology*, vol. 185, no. 5, pp. 1485–1491, 2003.

[2] C. W. Gardiner, *Handbook of stochastic methods for physics, chemistry and the natural sciences*, vol. 13 of *Springer Series in Synergetics*. Berlin: Springer-Verlag, third ed., 2004.

[3] G. Tkačik and W. Bialek, "Diffusion, dimensionality, and noise in transcriptional regulation," *Physical Review E*, vol. 79, pp. 051901–, 05 2009.

[4] D. H. Lenz, K. C. Mok, B. N. Lilley, R. V. Kulkarni, N. S. Wingreen, and B. L. Bassler, "The small rna chaperone hfq and multiple small rnas control quorum sensing in *Vibrio harveyi* and *Vibrio cholerae*," *Cell*, vol. 118, pp. 69–82, 7 2004.

[5] E. Levine, Z. Zhang, T. Kuhlman, and T. Hwa, "Quantitative characteristics of gene regulation by small rna," *PLoS Biology*, vol. 5, p. e229, 08 2007.

[6] M. Thattai and A. van Oudenaarden, "Intrinsic noise in gene regulatory networks," *Proceedings of the National Academy of Sciences*, vol. 98, no. 15, pp. 8614–8619, 2001.

[7] D. Jost, A. Nowojewski, and E. Levine, "Regulating the many to benefit the few: role of weak small rna targets," *Biophysical Journal*, vol. 104, pp. 1773–1782, 4 2013.

[8] C. P. Bahl, R. Wu, J. Stawinsky, and S. A. Narang, "Minimal length of the lactose operator sequence for the specific recognition by the lactose repressor," *Proceedings of the National Academy of Sciences*, vol. 74, no. 3, pp. 966–970, 1977.

[9] U. Alon, *An introduction to systems biology: Design principles of biological circuits*. Boca Raton: Chapman and Hall/CRC, 2006.

[10] S.-W. Teng, Y. Wang, K. C. Tu, T. Long, P. Mehta, N. S. Wingreen, B. L. Bassler, and N. P. Ong, "Measurement of the copy number of the master quorum-sensing regulator of a bacterial cell," *Biophysical Journal*, vol. 98, pp. 2024–2031, 5 2010.

[11] S.-W. Teng, J. N. Schaffer, K. C. Tu, P. Mehta, W. Lu, N. P. Ong, B. L. Bassler, and N. S. Wingreen, "Active regulation of receptor ratios controls integration of quorum-sensing signals in *Vibrio harveyi*," *Molecular Systems Biology*, vol. 7, 05 2011.

[12] T. M. Cover and J. A. Thomas, *Elements of information theory*. New York, NY, USA: Wiley-Interscience, 1991.

[13] G. Tkačik, C. G. Callan, and W. Bialek, "Information flow and optimization in transcriptional regulation," *Proceedings of the National Academy of Sciences*, vol. 105, no. 34, pp. 12265–12270, 2008.

[14] S. Arimoto, “An algorithm for computing the capacity of arbitrary discrete memoryless channels,” *Information Theory, IEEE Transactions on*, vol. 18, pp. 14–20, 01 1972.

[15] G. Tkačik, J. Callan, Curtis G., and W. Bialek, “Information capacity of genetic regulatory elements,” *Physical Review E*, vol. 78, 07 2008.
